# Stage-to-Stage Changes in Heart Rate–Power Output Coupling During Cycling Stage Races: Associations with Race-Derived Performance and Stage Context

**DOI:** 10.3390/sports14090409

**Published:** 2026-09-16

**Authors:** Jose Luis Sánchez-Jiménez, Alejandro Javaloyes, Mikel Zabala, Manuel Moya-Ramón, Manuel Mateo-March

**Affiliations:** 1 Research Group in Sports Biomechanics (GIBD), Department of Physical Education and Sports, University of Valencia, 46010 Valencia, Spain; jose.l.sanchez-jimenez@uv.es; 2 Sports Research Centre, Department of Sport Sciences, Miguel Hernández University, 03202 Elche, Spain; mmoya@umh.es (M.M.-R.); manuel.mateom@umh.es (M.M.-M.); 3 Sport and Health University Research Institute (iMUDS), Department of Physical Education and Sport, University of Granada, 18071 Granada, Spain; mikelz@ugr.es

**Keywords:** durability, heart rate–power output coupling, stage racing, internal–external load, mixed-effects modelling

## Abstract

This study examines whether model-derived heart rate–power output (HR–PO) parameters changed across consecutive stages of elite under-23 stage races and whether their longitudinal trajectories were associated with race-derived performance characteristics and stage context. Race files from the 2025 Tour de l’Avenir and Giro Next Gen were analysed, comprising 127 valid stage observations from 23 cyclist–race cases and 19 unique cyclists. For each stage, τ_del_, τ_rec_, and τ_max_ were estimated from an HR–PO model, and longitudinal changes were analysed using linear mixed-effects models. The estimated decrease in τ_del_ across race progression was not statistically significant after FDR correction across the three parameters (−1.33 s, 95% CI −2.45 to −0.20, unadjusted *p* = 0.021; q = 0.063), whereas τ_rec_ (0.68 s, 95% CI −6.58 to 7.93, *p* = 0.855) and τ_max_ (−0.96 s, 95% CI −2.10 to 0.19, *p* = 0.101) showed no systematic change. The τ_del_ trajectory differed between Avenir and Giro (race-by-progression interaction, *p* = 0.027) and was attenuated after exclusion of the most mountainous stages (−0.85 s, 95% CI −2.56 to 0.85, *p* = 0.327). In exploratory secondary analyses, higher race-derived PO at 5 s and 5–30 min (*p* ≤ 0.006, q ≤ 0.043) and better 5 min durability (*p* < 0.001, q = 0.007) were associated with a more negative τ_rec_ trajectory; however, cyclist-cluster bootstrap confidence intervals excluded zero only for the 5 s and 5 min PO interactions, and no association was retained for τ_del_ or τ_max_. In stage-context analyses, higher normalized power relative to critical power was associated with higher τ_rec_ (8.09 s per 10 percentage points; *p* = 0.001, q = 0.044), whereas greater low-intensity exposure was associated with lower τ_max_ (−0.82 s per 10 percentage points relative to moderate intensity; *p* = 0.003, q = 0.045); no stage-context association was retained for τ_del_. Overall, the estimated decrease in τ_del_ did not reach statistical significance after FDR correction and was sensitive to race context and the exclusion of the most mountainous stages, whereas τ_rec_ showed no systematic longitudinal change but varied in association with race-derived PO, durability, and relative stage intensity. All secondary associations are hypothesis-generating and require independent validation.

## 1. Introduction

Road cycling performance is not determined solely by the power output (PO) that cyclists can produce under fresh conditions, but also by their ability to sustain high PO after substantial prior work [1,2]. This capacity, commonly referred to as durability [3], has emerged as an important performance-related factor of competitive cycling performance, particularly in events characterized by prolonged duration, repeated high-intensity efforts, and large accumulated workloads [2,4]. Previous research in professional cyclists has shown that mean maximal power (MMP) progressively declines as prior work accumulates, and that higher-level cyclists are better able to attenuate this decline under fatigued conditions [1,5]. Moreover, recent evidence suggests that the intensity of prior work, and not only its total amount, contributes to the subsequent reduction in the power–duration relationship, with work performed above critical power (CP) being especially relevant for fatigue-related performance impairment [5,6].

Most durability research has focused either on acute fatigue responses within a single session or race [6,7], or on maximal power profiles derived from long-term datasets without specifically examining how responses evolve across consecutive race days [2,8]. However, stage races impose a different competitive context, in which cyclists must repeatedly perform after incomplete recovery and accumulated demands from previous days [9]. Previous stage-race studies have shown that performance differences between cyclists become more evident when fatigue and race progression are considered [10]. During a Grand Tour, WorldTour cyclists displayed greater durability than ProTeam cyclists as the race progressed from the first to the third week and when MMP was assessed after accumulated work within stages [10]. In addition, heart rate (HR)-based analyses of professional stage races have shown that internal workload responses vary with race duration and race progression, with lower mean and maximal HR, reduced time at high intensity, and lower daily training impulse (TRIMP) values during longer stage races and later race weeks [11]. These findings suggest that examining how cyclists respond across consecutive race days may provide relevant information about performance-related characteristics in stage racing. However, single-domain markers may provide only a partial interpretation of the cyclist’s response to stage-race demands. External-load variables such as PO describe the mechanical work performed, whereas internal-load variables such as HR reflect the cardiovascular response to that work. Therefore, integrating HR and PO may provide information beyond isolated measures of either signal by characterizing the temporal coupling between external work and the corresponding cardiovascular response.

Beyond absolute HR or PO values, the temporal dynamics between both signals may provide additional insight into the cyclist’s physiological response to exercise [12,13]. HR kinetics are not instantaneous, but depend on exercise intensity, time, fatigue-related processes, and the individual’s cardiovascular condition [14,15]. Therefore, HR at a given time point should be interpreted as a dynamic response to current and recent PO history, rather than as an isolated marker of internal load [12,16]. This is particularly relevant in road cycling, where the intermittent and stochastic nature of race demands produces repeated fluctuations in PO that require continuous cardiovascular adjustment [13]. Recent field-based approaches have modelled heart rate–power output (HR–PO) coupling using response-time parameters that describe the temporal weighting of current and preceding PO in relation to HR [12,16]. These parameters summarize features of the fitted HR–PO relationship, including the delayed and decaying influence of previous PO, but should not be interpreted as direct measurements of physiological kinetics. Previous work has reported associations between these model-derived parameters and endurance performance [12,13], supporting their use as field-based descriptors of HR–PO dynamics.

Therefore, the primary aim of this study was to examine whether model-derived HR–PO parameters changed across race progression during cycling stage races. Secondary aims were to examine whether their longitudinal trajectories were associated with race-derived PO and durability, and whether stage-level variation in these parameters was associated with stage-context characteristics. We hypothesized that race progression would be associated with shorter model-derived HR–PO response times, reflected by decreases in τ_del_, τ_rec_, and τ_max_.

## 2. Materials and Methods

### 2.1. Participants

Race data from male under-23 (U23) road cyclists competing in the 2025 editions of the Tour de l’Avenir and Giro Next Gen were screened for inclusion. Only cyclist–race cases with an official final race classification were included. Both races comprised eight consecutive race days, including time-trial, flat, and mountain stages. The Tour de l’Avenir covered 770 km with 14,028 m of elevation gain, whereas the Giro Next Gen covered 1029 km with 13,500 m of elevation gain. Prologues and individual time trials were not included in the HR–PO modelling because of their shorter duration and distinct race demands, but were retained for cumulative prior-load calculations. Accordingly, stages 2–7 were analysed for the Tour de l’Avenir, whereas the stage-1 prologue and stage-8 individual time trial were not modelled. For the Giro Next Gen, stages 2–8 were analysed, whereas the stage-1 individual time trial was not modelled.

**Figure 1:**
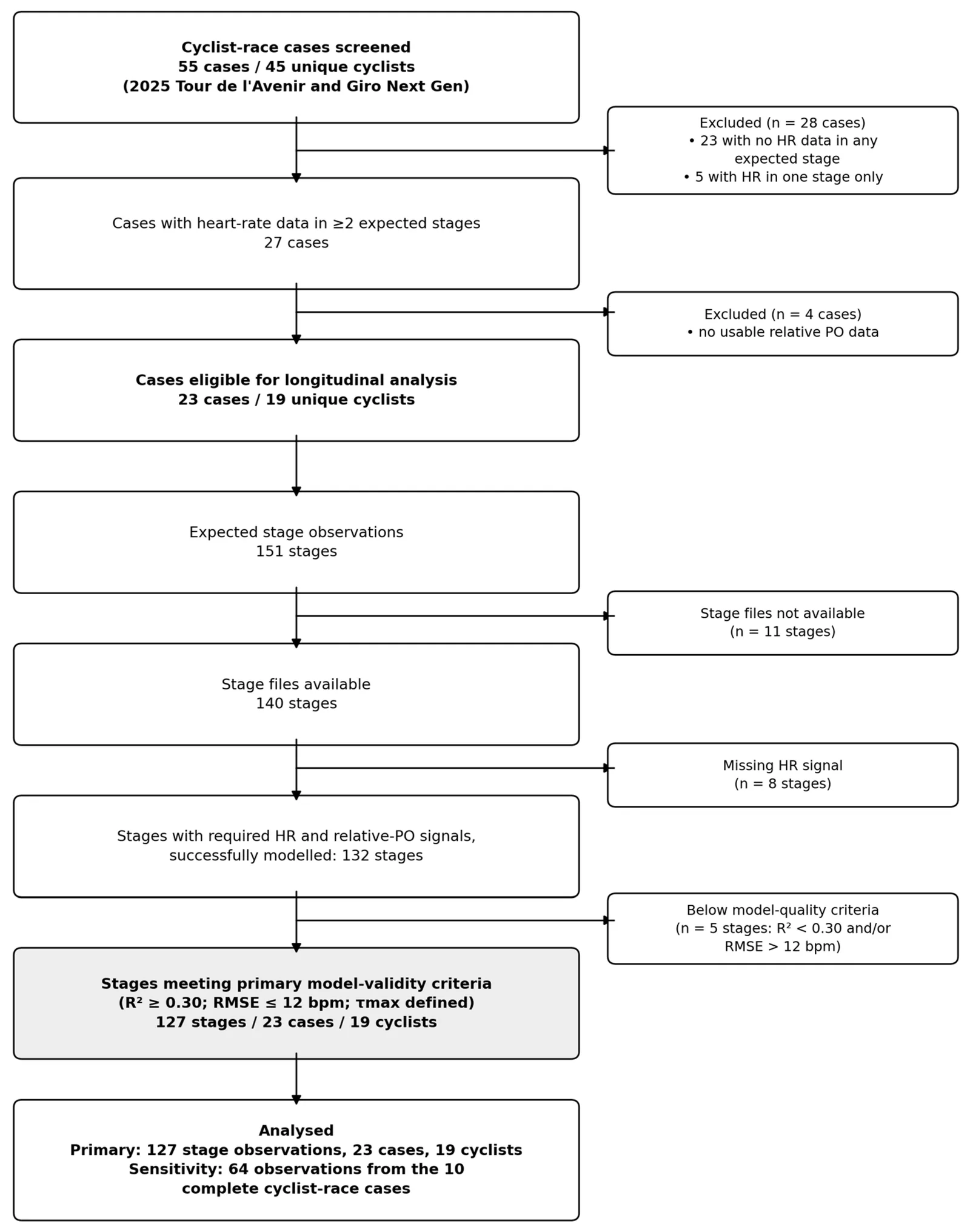
Flow of cyclist–race cases and stage files through screening, HR–PO modelling, and analysis.

A cyclist–race case was included in the longitudinal analyses when at least two eligible stages yielded valid HR–PO models. Of the 55 initially available cyclist–race cases, corresponding to 45 unique cyclists, 23 cyclist–race cases from 19 unique cyclists met the inclusion criteria. Across these cases, 151 stage observations were expected, 140 stage files were available, 132 contained the required HR and relative PO signals and were successfully modelled, and 127 met the predefined stage-level validity criteria and were included in the analyses. Ten cyclist–race cases had valid models for all expected stages and were retained for the complete case sensitivity analysis. The flow of cyclist–race cases and stage files is shown in Figure 1, and characteristics of the final analytical sample are presented in Table 1. This study analysed publicly available data; therefore, formal ethical approval and individual informed consent were not required [17]. All procedures were conducted in accordance with recognized ethical standards and the principles of the Declaration of Helsinki. Race files were obtained retrospectively from publicly accessible cyclist profiles and downloaded in their original .fit format. Files were pseudonymized by a single researcher before analysis by replacing cyclist identities with internal identification codes, and all subsequent data processing and statistical analyses were conducted using pseudonymized data.

### 2.2. Data Acquisition

Race files, downloaded retrospectively from publicly accessible Strava profiles, were recorded at 1 Hz using cyclists’ own power meters and chest-strap HR monitors. Power data were obtained from commercially available power meters, such as SRM (SRM GmbH, Jülich, Germany), Shimano (Shimano Inc., Sakai, Osaka, Japan), Power2Max (Saxonar GmbH, Waldhufen, Germany), or Quarq (SRAM LLC, Spearfish, SD, USA), whereas HR data were recorded using compatible chest-strap monitors from manufacturers such as Garmin (Garmin International, Inc., Olathe, KS, USA), Polar (Polar Electro Oy, Kempele, Finland), or Wahoo (Wahoo Fitness, LLC, Atlanta, GA, USA). Device models were not systematically identified for individual cyclist–race cases. Raw files were imported and visually inspected to verify data completeness, signal continuity, and the presence of the required variables, including PO and HR. A scripted quality-control procedure was subsequently applied using predefined validity ranges: HR 40–220 beats·min^−1^, absolute PO 0–1800 W, relative PO 0–30 W·kg^−1^, and non-negative accumulated relative work. Values outside these ranges were treated as missing. Isolated missing values were linearly interpolated using a five-sample limit in each direction, and observations remaining incomplete were excluded. HR and PO were recorded on the same 1-Hz time base and were not resampled or smoothed. Among the 132 stage files modelled, only two samples in one file required interpolation, no observations were removed during cleaning, and none of the 127 valid stage observations required interpolation or row deletion. Files lacking a required signal were not modelled. Device-specific calibration procedures were not available and could not be independently verified. Body mass was obtained from ProCyclingStats and a single value was applied to all stages within each cyclist–race case.

### 2.3. HR–PO Modelling Framework

HR–PO coupling was modelled using an adapted analytical framework based on the approach proposed by de Leeuw et al. [12,16]. The model assumes that HR at a given time point is influenced not only by the current PO, but also by the PO produced during the preceding 180 s. Accordingly, HR was modelled as a weighted function of current and prior relative PO, as follows (Equation (1)):

(1) $$H\left(t\right)=H_{0}+\sum_{j=0}^{j=180}h\left(-j\right)\cdot P\left(t-j\right)$$

where $H\left(t\right)$ is the HR at time t, $P\left(t\right)$ represents the PO at time t, and $H_{0}$ corresponds to the estimated HR after PO has been equal to 0 for 180 s. The function $h\left(-j\right)$ represents the weight assigned to the PO produced *j* seconds before time *t*, thereby describing how previous efforts contribute to the current HR response. As in the original framework, the primary lookback window was set at 180 s [12,16]. In the present implementation, $P\left(t\right)$ corresponded to relative PO (W·kg^−1^), allowing the HR–PO relationship to be modelled independently of differences in body mass between cyclists.

The weighting function *h* was defined to represent two physiological processes involved in the HR response to changes in PO. First, HR does not respond instantaneously to changes in exercise intensity but shows a short delay after changes in PO (τ_del_). Second, the influence of previous efforts progressively decays over time, such that more distant efforts contribute less to the current HR response (τ_rec_). In accordance with previous study [12], these two processes were represented by the following differential equation (Equation (2)):

(2) $$\frac{dh\left(t\right)}{d\left(-t\right)}=-\frac{1}{\tau_{rec}}h\left(t\right)+\frac{1}{\tau_{del}}f\left(t\right)$$

where τ_rec_ represents the recovery time constant, describing how rapidly the influence of previous exercise decays, and τ_del_ represents the delay time constant, describing the timescale over which the delayed HR response remains relevant. The delayed component was modelled using a sigmoid function (Equation (3)) [12,18]:

(3) $$f\left(t\right)=\frac{e^{t/\tau_{del}}}{1+e^{t/\tau_{del}}}$$

where τ_del_ determines the timescale over which the delay in the HR response remains relevant. Thus, lower τ_del_ values reflect a shorter delay in the HR response to changes in PO, whereas higher values reflect a more prolonged delayed response.

The resulting analytical solution for the weighting function was (Equation (4)) [12]:

(4) $$h\left(t\right)=2h\left(0\right)\cdot \frac{e^{t/\tau_{rec}}}{1+e^{t/\tau_{del}}}$$

where *h*(0) is the value of the weighting function at *t* = 0. This function represents the combined delayed and decaying influence of previous PO on current HR. In the present implementation, this analytical weighting function was fitted directly to the HR–PO time series. Thus, no empirical free-lag kernel was first estimated and subsequently approximated by the analytical function. Instead, the analytical kernel was imposed a priori, and its parameters were estimated by optimizing the agreement between modelled and observed HR. Specifically, *H*_0_, *h*(0), τ_del_, and τ_rec_ were estimated simultaneously using bounded nonlinear least-squares optimization. Parameters were estimated using bounded nonlinear least squares with the trust-region-reflective algorithm, a soft-L1 loss function, and a maximum of 5000 function evaluations. Parameter bounds were *H*_0_ = 30–180 beats·min^−1^, *h*(0) = 0–20, τ_del_ = 0.1–60 s, and τ_rec_ = 1–300 s. These broad bounds were used as analytical constraints to exclude implausible or degenerate solutions while allowing substantial between-stage variability. Starting values were the 5th percentile of observed HR for *H*_0_, 0.20 for *h*(0), 5.0 s for τ_del_, and 40.0 s for τ_rec_. A single deterministic starting point was used; no multiple-start optimization procedure was applied. Practical parameter uncertainty was additionally assessed using a moving-block bootstrap with 100 resamples per stage (block length = 900 s; random seed = 42), with the model refitted in each resample and percentile 95% confidence intervals calculated for τ_del_, τ_rec_, and τ_max_.

In addition to τ_del_ and τ_rec_, τ_max_ was calculated as (Equation (5)) [12]:

(5) $$\tau_{max}=\tau_{del}\cdot log\left(\frac{\tau_{rec}-\tau_{del}}{\tau_{del}}\right)$$

This parameter represents the time lag at which prior PO has the greatest estimated influence on current HR and was derived from τ_del_ and τ_rec_, rather than fitted independently. Throughout this study, τ_del_, τ_rec_, and τ_max_ were treated as model-derived descriptors of HR–PO coupling rather than as directly measured physiological time constants.

In the present study, the HR–PO framework was adapted to stage-level competition files. Unlike the original weekly approach, models were fitted separately for each stage because the aim was to describe stage-to-stage changes in HR–PO coupling during stage races, rather than to estimate weekly fitness status or predict laboratory performance. For each stage, HR and relative PO data were aligned at 1 Hz, and lagged relative-PO matrices were constructed using the current second and the preceding 180 s; the first 180 s of each stage were used only to provide lag history and were not included as modelled observations. A pre-specified rule allowed a random subset of 30,000 observations (random seed = 42) to be used for parameter optimization when this number was exceeded. This rule was never triggered, as the largest stage contained 18,825 modelled observations; therefore, all reported parameter estimates were obtained from the complete available stage series. Model fitting was performed directly on the analytical HR–PO model. Therefore, the R^2^ reported in the present study refers to the goodness-of-fit of the final analytical model against the observed HR time series for the full stage. It does not refer to the R^2^ of an empirical kernel estimation step or to a train–test validation of an unconstrained lag-weight regression. No random 80/20 train–test split was applied. Valid stage-level outputs required successful model convergence, R^2^ ≥ 0.30, RMSE ≤ 12 beats·min^−1^, and finite positive τ_max_ (requiring τ_rec_ > 2·τ_del_). Because these thresholds represent analytical conventions rather than validated cut-offs, a stricter criterion of R^2^ ≥ 0.50 combined with RMSE ≤ 12 beats·min^−1^ was examined as a sensitivity analysis. Model fit statistics and parameter estimates were stored for subsequent quality control and statistical analysis. Only valid full-stage HR–PO model outputs were included in the primary longitudinal analyses.

### 2.4. Performance-Profile Extraction and Derived Variables

Race-derived PO (W·kg^−1^) profiles were calculated for 5 s, 30 s, 1 min, 5 min, 10 min, 20 min, and 30 min for each cyclist–race case. The highest unrestricted race-derived MMP recorded across the race was retained as the reference value.

Durability (%) was quantified from the change in PO after increasing amounts of accumulated within-stage work. For each duration, the highest mean PO available across all stages of the race after accumulated-work thresholds of 10, 20, 30, 40, 50, and 60 kJ·kg^−1^ was expressed as a percentage change from the unrestricted race-derived reference value. The unrestricted reference was assigned a value of 0% at 0 kJ·kg^−1^. Percentage-change values across 0–60 kJ·kg^−1^ were integrated using the trapezoidal rule and divided by the 60-kJ·kg^−1^ work range, yielding a normalized area-under-the-curve value expressed as a percentage. Thus, 0% indicates no deterioration in PO across the accumulated-work range, whereas increasingly negative values indicate greater deterioration; higher (less negative) values therefore indicate better preservation of PO. Calculation of durability required values at both 0 and 60 kJ·kg^−1^. Accumulated-work thresholds were nested, meaning that the same maximal effort could contribute to more than one successive threshold. Threshold-specific values were therefore not analysed as independent performance variables; instead, the normalized area under the curve (AUC) provided a single durability value for each cyclist–race case and duration.

Critical power (CP, W·kg^−1^) and W′ (J·kg^−1^) were modeled from the relative maximum record power profile (W·kg^−1^) (2, 5 and 12 min) with the inverse of time model (1/t); that is, plotting the MMP for each duration against the inverse of the time (t), with CP corresponding to the y-intercept and W′ corresponding to the slope, representing the finite amount of work that can be performed above CP [19]. Model fit was assessed using R^2^ and the standard error of the estimate (SEE).

### 2.5. Stage-Context Descriptors

Stage-context descriptors were calculated for each cyclist at each stage. Stage profile was characterized by stage duration, elevation gain (m), and accumulated work (kJ·kg^−1^). Normalized power (NP) was calculated from the 30 s rolling mean PO using the fourth-power procedure and was expressed relative to individual CP (%CP). PO variability was quantified as the coefficient of variation (CV, %). Coasting exposure was calculated as the percentage of stage time with PO between 0 and 1 W.

Power-intensity distribution was quantified relative to CP as low-intensity (<70% CP; LIT), moderate-intensity (70–100% CP; MIT), and high-intensity (>100% CP; HIT) exposure and expressed as a percentage [20,21]. HR-intensity distribution was defined relative to the maximum HR observed for each cyclist across the race (HR_max_) and summarized as low (50–69% HR_max_), moderate (70–79% HR_max_), and high (80–100% HR_max_) exposure [22,23]. HR-zone shares were expressed relative to the total time accumulated between 50% and 100% HR_max_, such that time below 50% HR_max_ was not assigned to any of the three components. The ratio between stage NP (W) and mean HR (beats·min^−1^) was calculated as an external-to-internal load ratio.

Cumulative prior mechanical work and Edwards’ TRIMP were additionally calculated across all preceding race stages, including stages not included in HR–PO modelling when applicable. Edwards’ TRIMP was calculated from time accumulated in five HR zones (50–59%, 60–69%, 70–79%, 80–89%, and 90–100% HR_max_), weighted by factors of 1–5, respectively [22,23].

### 2.6. Statistical Analysis

Race progression was normalized separately within each race from 0 (first analysed stage) to 1 (final analysed stage). Unless otherwise specified, stage-level linear mixed-effects models included crossed random intercepts for cyclist–race case and shared race-stage context and were estimated using restricted maximum likelihood (REML). Model convergence and variance components were inspected. Descriptive characteristics were summarized as mean ± SD or median [IQR], as appropriate. All tests were two-sided, and effect estimates are reported with 95% confidence intervals. Unless otherwise specified, model-based 95% confidence intervals were Wald-type, whereas bootstrap confidence intervals were percentile-based. Mixed-effects *p* values and Wald confidence intervals were based on asymptotic (z) inference; small-sample denominator degrees-of-freedom corrections were not applied, and cluster-bootstrap resampling was used instead to assess the influence of the limited number of clusters. All analyses were performed in Python (Version 3.13.15) using Google Colaboratory, with statistical modelling conducted primarily using statsmodels and SciPy and data processing using NumPy and pandas.

In the longitudinal analysis, separate mixed-effects models were fitted for τ_del_, τ_rec_, and τ_max_, with race progression and race as fixed effects. Because progression was scaled from 0 to 1, its coefficient represents the estimated beginning-to-end change in each parameter. A random slope for progression was evaluated but not retained because the estimated covariance structure was near-boundary.

Potential non-linearity in τ_del_ was examined by adding a quadratic progression term and comparing linear and quadratic models fitted by maximum likelihood using a likelihood-ratio test and the Akaike information criterion (AIC). Differences between races were assessed using a race-by-progression interaction followed by race-specific models. Robustness of the primary τ_del_ estimate was examined in the 10 complete cyclist–race cases, under stricter model-quality criteria (R^2^ ≥ 0.50 and RMSE ≤ 12 beats·min^−1^), after exclusion of the most mountainous stages, using 2000 cyclist–race-case cluster-bootstrap resamples, and using a Gaussian generalized estimating equation with a first-order autoregressive [AR(1)] working correlation structure for serial dependence within cyclist-race cases.

Sensitivity of the τ_del_ trajectory to model quality and stage characteristics was examined by modelling R^2^, RMSE, and MAE across race progression and by re-estimating τ_del_ after adjustment for either R^2^ or RMSE together with PO coefficient of variation and coasting exposure, or for elevation gain, stage duration, and stage work. Cumulative prior work was additionally examined, with multicollinearity assessed using variance inflation factors (VIFs); because it was strongly collinear with race progression, the corresponding coefficients were not interpreted as independent effects. Residual distribution and heteroscedasticity were assessed using the Shapiro–Wilk and Breusch–Pagan tests, respectively.

Sensitivity to the lookback specification was assessed by re-estimating the HR–PO model using 120, 180, and 240 s windows and repeating the τ_del_ analysis in stages valid across all three specifications. Agreement with the 180 s specification was quantified using absolute-agreement intraclass correlation coefficients (ICCs). Within-stage stability was examined using split-half analyses, retaining the preceding 180 s as lag history for the second half. Analyses were restricted to stages valid in both halves, and agreement was assessed using absolute and relative differences, Spearman correlations, Lin’s concordance correlation coefficient, and absolute-agreement ICCs. Time-resolved observed-versus-modelled HR and residual trajectories were also examined for objectively selected low- and high-elevation stage contexts, using the cyclist-stage observation with model-fit statistics closest to the context median. As a multiplicity sensitivity analysis, Benjamini–Hochberg-adjusted q values were additionally calculated across the three longitudinal parameter tests.

Performance-related analyses. Associations between race-derived PO, durability, and longitudinal trajectories of model-derived HR–PO parameters were examined at seven durations (5 s, 30 s, 1 min, 5 min, 10 min, 20 min, and 30 min). PO was expressed relative to body mass (W·kg^−1^), and durability as a percentage. For each duration, both variables were standardized across cyclist-race cases (z scores) and entered simultaneously into the model. Their association and collinearity were assessed using Spearman correlations and VIFs.

Separate models were fitted for τ_del_, τ_rec_, and τ_max_ at each duration. Fixed effects included race progression, race, the race-by-progression interaction, PO, durability, and the interaction of progression with each performance variable. Progression-by-performance coefficients therefore represent the additional beginning-to-end change in the corresponding HR–PO parameter associated with a 1-SD higher PO or durability value.

Performance-related analyses were considered secondary and exploratory. Benjamini–Hochberg false-discovery-rate correction was applied jointly across the 42 progression-by-performance interactions (three parameters × seven durations × two performance variables). Robustness was assessed using leave-one-cyclist-out analyses and 2000 unique-cyclist cluster-bootstrap resamples. Because race-derived PO and durability were defined at the cyclist–race-case level whereas the HR–PO parameters varied at the stage level, the progression-by-performance terms represent cross-level interactions. As the random slope for progression was not retained, model-based standard errors for these terms were estimated against the stage-level residual variance and are therefore expected to be anticonservative. Model-based Wald inference is consequently reported for completeness, whereas the unique-cyclist cluster-bootstrap intervals, which resample at the level at which the performance variables vary, were considered the primary basis for inference within this analysis family. In leave-one-cyclist-out analyses, all race participations of the excluded cyclist were removed simultaneously. For bootstrap analyses, unique cyclists were resampled with replacement while retaining all observations and race participations from each sampled cyclist, and percentile 95% confidence intervals were calculated.

As a sensitivity analysis, final HR–PO parameter values were modelled for cyclist–race cases with valid observations at both 0% and 100% race progression as a function of the corresponding baseline value, PO, durability, and race, using cluster-robust standard errors at the unique-cyclist level. Spearman correlations between baseline values and beginning-to-end changes were additionally examined to assess baseline dependency and potential regression to the mean. Within each parameter–performance construct, Benjamini–Hochberg false-discovery-rate correction was applied across the seven performance durations.

In the stage-context analyses associations between stage characteristics and model-derived HR–PO parameters were examined using the same valid stage observations. Stage-context variables were organized into five complementary blocks: stage profile (elevation gain and stage work), power-signal structure (normalized power relative to critical power [NP, %CP], PO coefficient of variation, and coasting exposure), power-intensity distribution (LIT and HIT, with MIT as the reference component), HR-intensity distribution (low and high HR-zone exposure, with the moderate zone as the reference component), and the NP-to-mean-HR ratio as an external-to-internal load ratio.

Separate models were fitted for τ_del_, τ_rec_, and τ_max_ within each block. Fixed effects included race progression, race, and the race-by-progression interaction. Race progression was centred at the race midpoint for these analyses. Predictors were expressed as elevation gain per 100 m, stage work per 10 kJ·kg^−1^, NP, PO coefficient of variation, coasting, and intensity-distribution components per 10 percentage points, and NP-to-mean-HR ratio per 0.10 unit. For intensity-distribution models, coefficients represent the estimated difference associated with replacing 10 percentage points of the reference component with the corresponding intensity component while holding the other included component constant.

Stage duration was analysed separately from stage work because the two variables were strongly correlated (Spearman ρ = 0.90). The three stage-duration coefficients were treated as a separate multiplicity family and corrected using the Benjamini–Hochberg procedure. Cumulative prior work and cumulative prior Edwards’ TRIMP were not included in the stage-context models because of their strong dependence on race progression and were retained for collinearity assessment. Predictor collinearity was evaluated using VIFs.

Benjamini–Hochberg false-discovery-rate correction was applied jointly across the 30 stage-context tests (three parameters × 10 coefficients), with q < 0.05 defining associations retained after correction. Robustness was assessed using 2000 unique-cyclist cluster-bootstrap resamples, retaining both race participations together when a cyclist contributed to both races. Percentile bootstrap 95% confidence intervals and the percentage of resamples retaining the direction of the full-sample coefficient were calculated. Random-effect and residual variances, residual distribution, and heteroscedasticity were additionally inspected for these models.

## 3. Results

### 3.1. Sample Characteristics, Model Fit, and Parameter Stability

The analytical sample comprised 127 valid stage observations from 23 cyclist–race cases and 19 unique cyclists, including 53 observations from the Tour de l’Avenir and 74 from the Giro Next Gen across 13 shared race-stage contexts (Figure 1; Table 1). Across valid stages, median [IQR] τ_del_, τ_rec_, and τ_max_ were 6.8 [5.2–7.4] s, 44.2 [38.4–51.6] s, and 10.8 [9.8–12.2] s, respectively. Median model fit was R^2^ = 0.84 [0.79–0.88], with an RMSE of 7.1 [5.9–8.2] beats·min^−1^ and an MAE of 5.2 [4.4–6.1] beats·min^−1^. The three-point CP model showed a median R^2^ of 0.955 [0.859–0.993] and a median SEE of 0.166 [0.087–0.371] W·kg^−1^ across the 23 cyclist–race cases.

Model fit improved across race progression: R^2^ increased by 0.093 (95% CI [0.019, 0.167], *p* = 0.014), whereas RMSE decreased by 1.47 beats·min^−1^ (95% CI [−2.65, −0.29], *p* = 0.015) and MAE by 1.16 beats·min^−1^ (95% CI [−2.03, −0.29], *p* = 0.009). Representative time-resolved observed and modelled HR signals and residuals for low- and high-elevation stage contexts are shown in Supplementary Figure S1.

Whole-stage residual bias was small, but a systematic within-stage pattern was observed. Residual bias became more negative from the first to the second half of the stage in most files, with a median within-stage change of −4.13 beats·min^−1^ [IQR −6.87 to −1.00; Wilcoxon *p* < 0.001]. Because residuals were defined as modelled minus observed HR, this indicates the model’s increasing underestimation of HR as stages progressed, in a pattern consistent with cardiovascular drift that is not explicitly represented in the model.

Residuals from the primary τ_del_ model showed no clear departure from normality (Shapiro–Wilk *p* = 0.080), although heteroscedasticity was detected (Breusch–Pagan *p* = 0.009). The direction and magnitude of the progression estimate remained consistent in the cluster-bootstrap and AR(1) sensitivity analyses.

The longitudinal τ_del_ estimate was consistent across alternative lookback windows. The primary 180 s analysis included 127 valid stages. To ensure a like-for-like comparison across lookback specifications, the sensitivity analysis was restricted to the 126 stages that met the validity criteria with all three windows. Within this common-stage set, the estimated beginning-to-end changes were −1.43 s (95% CI [−2.24, −0.63], *p* < 0.001) with a 120 s window, −1.40 s (95% CI [−2.50, −0.30], *p* = 0.012) with 180 s, and −1.42 s (95% CI [−2.65, −0.19], *p* = 0.024) with 240 s. Stage-level τ_del_ estimates showed high agreement with the 180 s specification (ICC = 0.91 and 0.96 for 120 and 240 s, respectively; Supplementary Table S2).

In split-half analyses, 104 stages could be estimated in both halves and 100 met the validity criteria in both. Median τ_del_ was 6.79 s in the first half and 6.86 s in the second half (mean difference = −0.07 s), with modest agreement between half-stage estimates (ICC = 0.26). Agreement was similarly modest for τ_max_ (ICC = 0.24) and substantially lower for τ_rec_ (ICC = 0.01; Supplementary Table S1). The moving-block bootstrap indicated substantial uncertainty in individual stage-level estimates, with median 95% CI widths of 3.07 s for τ_del_, 21.22 s for τ_rec_, and 3.58 s for τ_max_, corresponding to 47.5%, 47.7%, and 31.4% of their respective point estimates; 100/100 bootstrap refits converged in all but one stage (minimum = 98).

### 3.2. Longitudinal Changes in Model-Derived HR–PO Parameters

In the primary mixed-effects models, τ_del_ decreased across race progression (B = −1.33 s, 95% CI [−2.45, −0.20], *p* = 0.021), whereas no clear longitudinal changes were observed for τ_rec_ (B = 0.68 s, 95% CI [−6.58, 7.93], *p* = 0.855) or τ_max_ (B = −0.96 s, 95% CI [−2.10, 0.19], *p* = 0.101). The τ_del_ trajectory was consistent with a linear change, as adding a quadratic progression term did not improve the model fit (quadratic term: B = −0.42 s, *p* = 0.817; likelihood-ratio *p* = 0.817; AIC = 455.9 vs. 457.8 for the linear and quadratic models, respectively). In the multiplicity sensitivity analysis, the Benjamini–Hochberg-adjusted q values were 0.063 for τ_del_, 0.855 for τ_rec_, and 0.152 for τ_max_.

The τ_del_ estimate remained negative across sensitivity analyses, although its magnitude and precision varied (Table 2). The estimated beginning-to-end change was −1.50 s (95% CI [−2.46, −0.54], *p* = 0.002) in the 10 complete cyclist–race cases, −1.40 s (95% CI [−2.50, −0.30], *p* = 0.012) under the stricter model-quality criteria, and −1.32 s (95% CI [−2.40, −0.25], *p* = 0.016) after excluding the stage with a τ_del_ estimate at the lower parameter bound. Cluster-bootstrap inference yielded a comparable estimate (B = −1.35 s, 95% CI [−1.98, −0.67]), while the AR(1) model also supported a negative progression effect (B = −1.05 s, 95% CI [−1.79, −0.31], *p* = 0.005).

**Figure 2:**
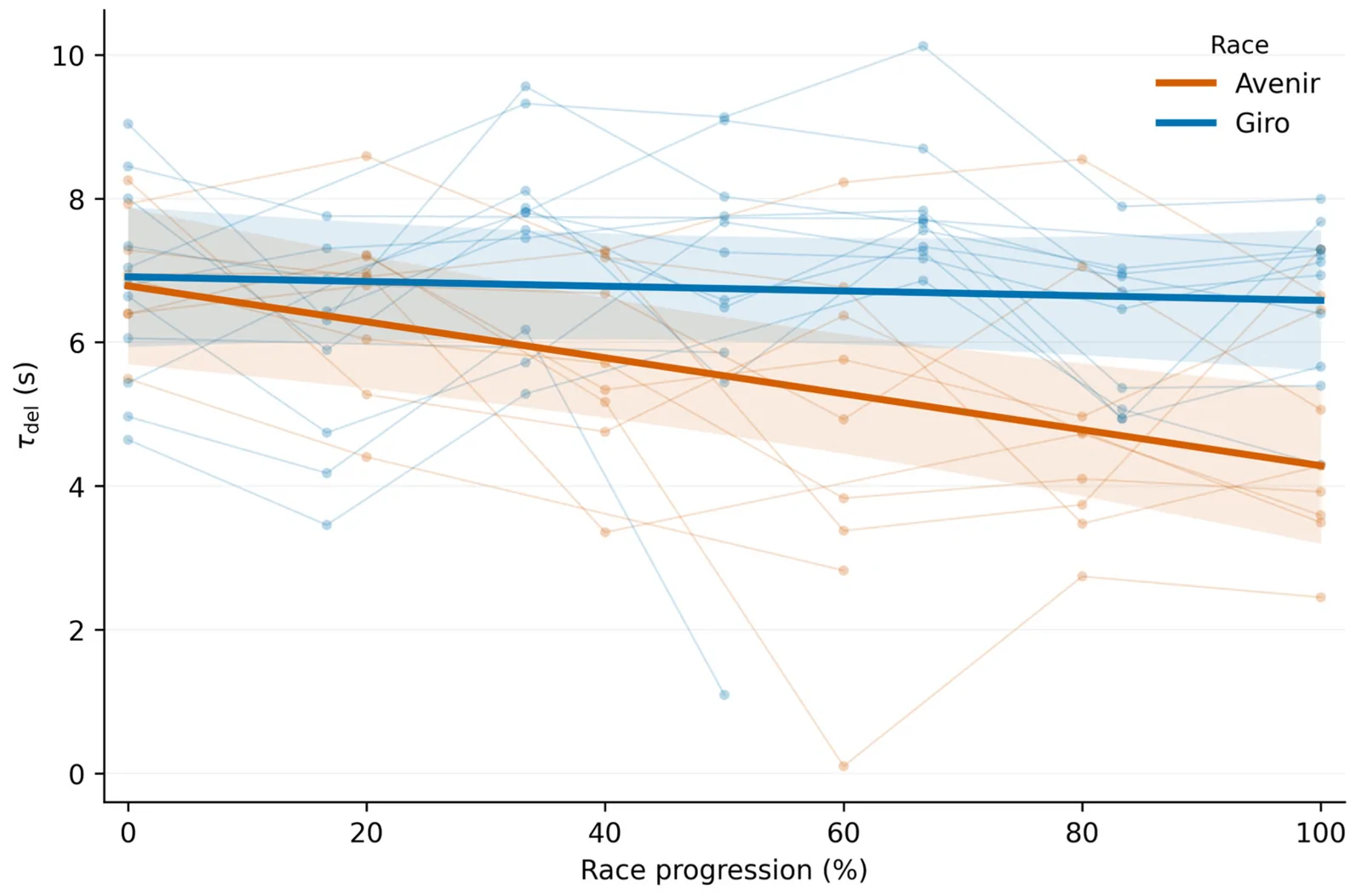
Individual and race-specific trajectories of τ_del_ across race progression. Thin lines and points represent individual cyclist-race-case observations. Thick lines represent fixed-effect predictions from the race-by-progression mixed-effects model, and shaded areas indicate the corresponding 95% confidence intervals for these fixed-effect predictions, not for the individual observations. Race progression is displayed from 0% (beginning) to 100% (end).

The magnitude of the τ_del_ change differed between races (race-by-progression interaction, *p* = 0.027). A decrease was observed in the Tour de l’Avenir (B = −2.48 s, 95% CI [−3.59, −1.37], *p* < 0.001), whereas the estimate was smaller and uncertain in the Giro Next Gen (B = −0.36 s, 95% CI [−1.89, 1.17], *p* = 0.644). Excluding the most mountainous stages attenuated the overall progression estimate (B = −0.85 s, 95% CI [−2.56, 0.85], *p* = 0.327; Table 2). Individual and race-specific τ_del_ trajectories are shown in Figure 2.

Adjustment for model quality and PO-signal structure produced similar progression estimates: B = −1.47 s (95% CI [−2.93, −0.01], *p* = 0.048) after adjustment for R^2^, PO coefficient of variation, and coasting exposure, and B = −1.40 s (95% CI [−2.67, −0.12], *p* = 0.032) after adjustment for RMSE, PO coefficient of variation, and coasting exposure. In contrast, adjustment for elevation gain, stage duration, and stage work attenuated the progression estimate (B = −0.94 s, 95% CI [−2.18, 0.31], *p* = 0.139).

### 3.3. Race Performance-Related Associations

PO and durability showed low collinearity across all durations (|Spearman ρ| ≤ 0.27; VIF ≤ 1.42). Because these variables were defined at the cyclist–race-case level, the progression-by-performance terms are cross-level interactions for which model-based inference is expected to be anticonservative; unique-cyclist cluster-bootstrap intervals are therefore reported alongside the model-based estimates and were used as the primary basis for inference. After FDR correction across the 42 progression-by-performance interactions, no association was retained for τ_del_ or τ_max_. For τ_rec_, higher PO was associated with a more negative longitudinal trajectory at 5 s (B = −10.93 s per SD, 95% CI [−16.58, −5.29], *p* < 0.001, q = 0.003), 5 min (B = −14.20 s per SD, 95% CI [−21.52, −6.88], *p* < 0.001, q = 0.003), 10 min (B = −9.00 s per SD, 95% CI [−14.20, −3.79], *p* < 0.001, q = 0.007), 20 min (B = −7.56 s per SD, 95% CI [−12.97, −2.15], *p* = 0.006, q = 0.043), and 30 min (B = −7.80 s per SD, 95% CI [−12.90, −2.70], *p* = 0.003, q = 0.023). The corresponding associations at 30 s (*p* = 0.020, q = 0.106) and 1 min (*p* = 0.018, q = 0.106) were also negative but were not retained after FDR correction.

**Figure 3:**
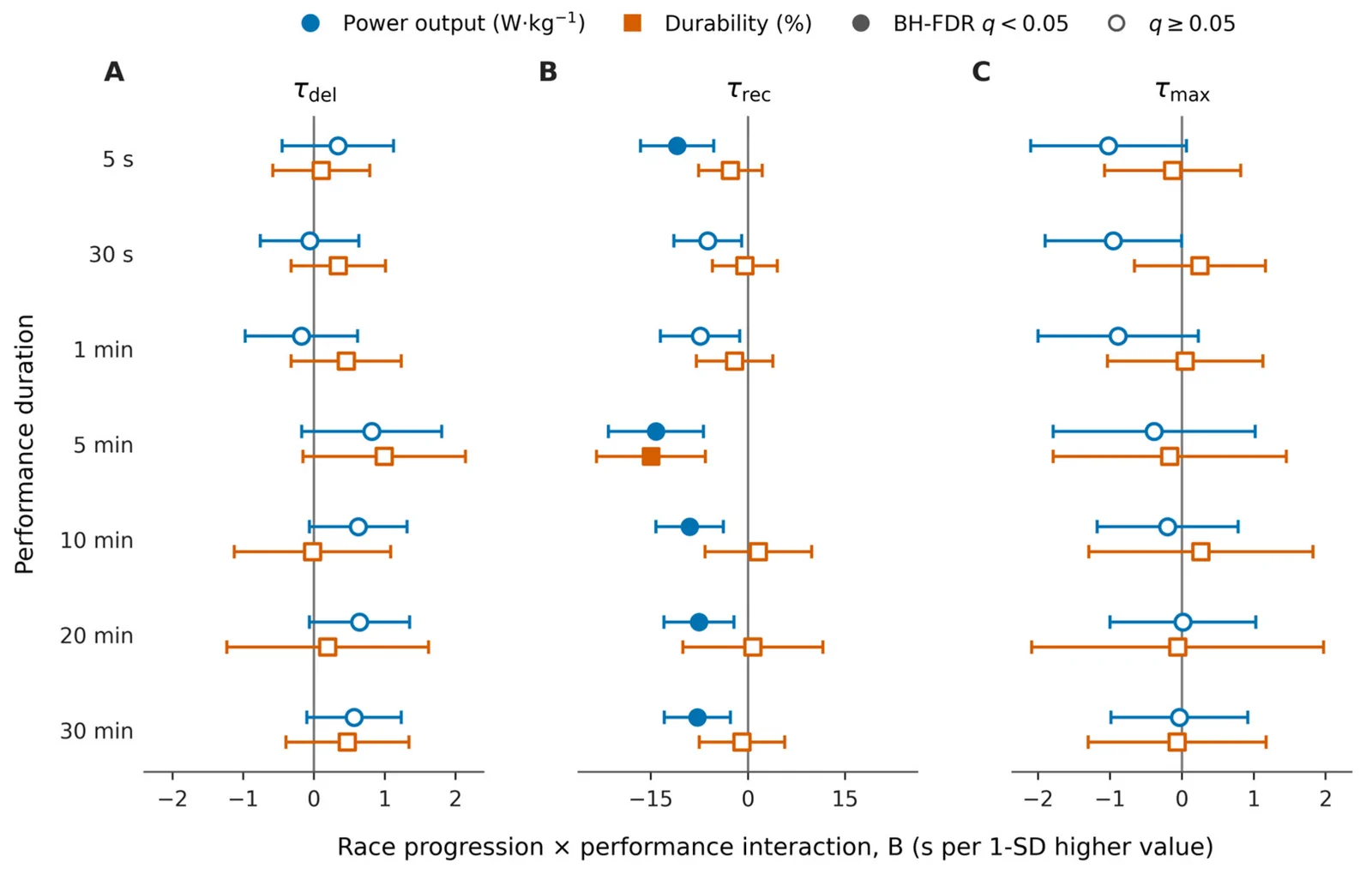
Associations of power output and durability with longitudinal changes in model-derived HR–PO parameters. Forest plots show progression-by-performance interaction estimates (**B**) and 95% confidence intervals for τ_del_ (**A**), τ_rec_ (**B**), and τ_max_ (**C**) across seven performance durations. Power output was expressed relative to body mass (W·kg^−1^), and durability (%) represents the normalized area under the curve of the percentage change in power output across 0–60 kJ·kg^−1^ of accumulated within-stage work. Estimates represent the additional beginning-to-end change in the corresponding HR–PO parameter associated with a 1 SD higher performance value. Filled markers indicate associations retained after Benjamini–Hochberg false-discovery-rate correction across all 42 progression-by-performance interactions (q < 0.05); open markers indicate q ≥ 0.05.

Durability showed a more duration-specific association with τ_rec_. Higher 5 min durability was associated with a more negative τ_rec_ trajectory after adjustment for 5 min PO (B = −14.96 s per SD, 95% CI [−23.35, −6.56], *p* < 0.001, q = 0.007), although the corresponding cluster-bootstrap interval included zero (−32.44 to 13.73 s per SD). No other durability association was retained after FDR correction (Figure 3; Supplementary Table S3).

Robustness analyses indicated consistent direction but substantially greater uncertainty than the model-based intervals. All five FDR-retained PO associations and the 5 min durability association remained negative in each of the 19 leave-one-cyclist-out analyses. However, cyclist-cluster bootstrap 95% CIs excluded zero for only two of these six associations: the 5 s (−23.07 to −1.11 s per SD) and 5 min (−34.19 to −0.52 s per SD) PO interactions. The 10, 20, and 30 min PO coefficients remained negative in 96.9%, 94.2%, and 91.8% of bootstrap resamples, respectively, and the 5 min durability coefficient was directionally consistent in all leave-one-cyclist-out analyses, but the corresponding bootstrap 95% CIs included zero. Accordingly, only the 5 s and 5 min PO interactions were supported by both FDR-corrected model-based inference and cluster-bootstrap inference, whereas the remaining associations were directionally consistent but not robustly established. By contrast, in the stage-context models, in which predictors varied at the stage level, model-based and bootstrap intervals were closely comparable, indicating that this discrepancy was specific to the cross-level performance terms.

The endpoint sensitivity analysis included 16 cyclist–race cases from 13 unique cyclists. Higher 5 s PO was associated with a higher final τ_del_ after adjustment for baseline τ_del_, durability, and race (B = 1.13 s per SD, 95% CI [0.38, 1.88], *p* = 0.007, q = 0.046). The 5 min PO association with final τ_rec_ remained nominally evident after adjustment for baseline τ_rec_, durability, and race (B = −7.42 s per SD, 95% CI [−14.43, −0.41], *p* = 0.040, q = 0.278). Baseline values were not associated with beginning-to-end changes in τ_del_ (*p* = 0.184), τ_rec_ (*p* = 0.122), or τ_max_ (*p* = 0.184), providing no clear evidence of regression to the mean (Supplementary Table S4).

### 3.4. Stage-Context Associations

Descriptive characteristics of the stage-context variables across the 127 valid stage observations are presented in Supplementary Table S5. All 15 stage-context mixed-effects models converged without near-zero random-effect variances, with acceptable collinearity across descriptor blocks (VIF = 1.08–4.58). Departures from residual normality were detected in 14 of the 15 stage-context models by the Shapiro–Wilk test, including all τ_rec_ and τ_max_ models and four of the five τ_del_ models (Supplementary Table S7). Therefore, model-based Wald confidence intervals were considered alongside percentile confidence intervals from 2000 unique-cyclist cluster-bootstrap resamples as a robustness analysis. After FDR correction across the 30 stage-context tests, higher NP relative to CP was associated with higher τ_rec_ (B = 8.09 s per 10 percentage points, 95% CI [3.11, 13.07], *p* = 0.001, q = 0.044). The bootstrap 95% CI also excluded zero ([2.44, 13.81]), with the coefficient retaining the same direction in 99.8% of 2000 cyclist-cluster resamples. Two additional τ_rec_ associations did not meet the FDR threshold: replacing 10 percentage points of MIT with HIT (B = 5.89 s, 95% CI [1.30, 10.48], *p* = 0.0118, q = 0.059) and replacing 10 percentage points of moderate HR-zone exposure with high HR-zone exposure (B = 2.37 s, 95% CI [0.61, 4.14], *p* = 0.008, q = 0.050). No other τ_rec_ association was retained after FDR correction. Residual diagnostics are reported in Supplementary Table S7.

For τ_max_, replacing 10 percentage points of MIT with LIT was associated with a lower value (B = −0.82 s, 95% CI [−1.36, −0.28], *p* = 0.003, q = 0.045). The bootstrap 95% CI was [−1.27, −0.40], and the coefficient remained negative in all 2000 resamples. Greater low-HR-zone exposure was also associated with lower τ_max_ before FDR correction (B = −0.56 s per 10 percentage points replacing moderate HR-zone exposure, 95% CI [−1.07, −0.06], *p* = 0.029, q = 0.124), whereas no other τ_max_ association was retained.

**Figure 4:**
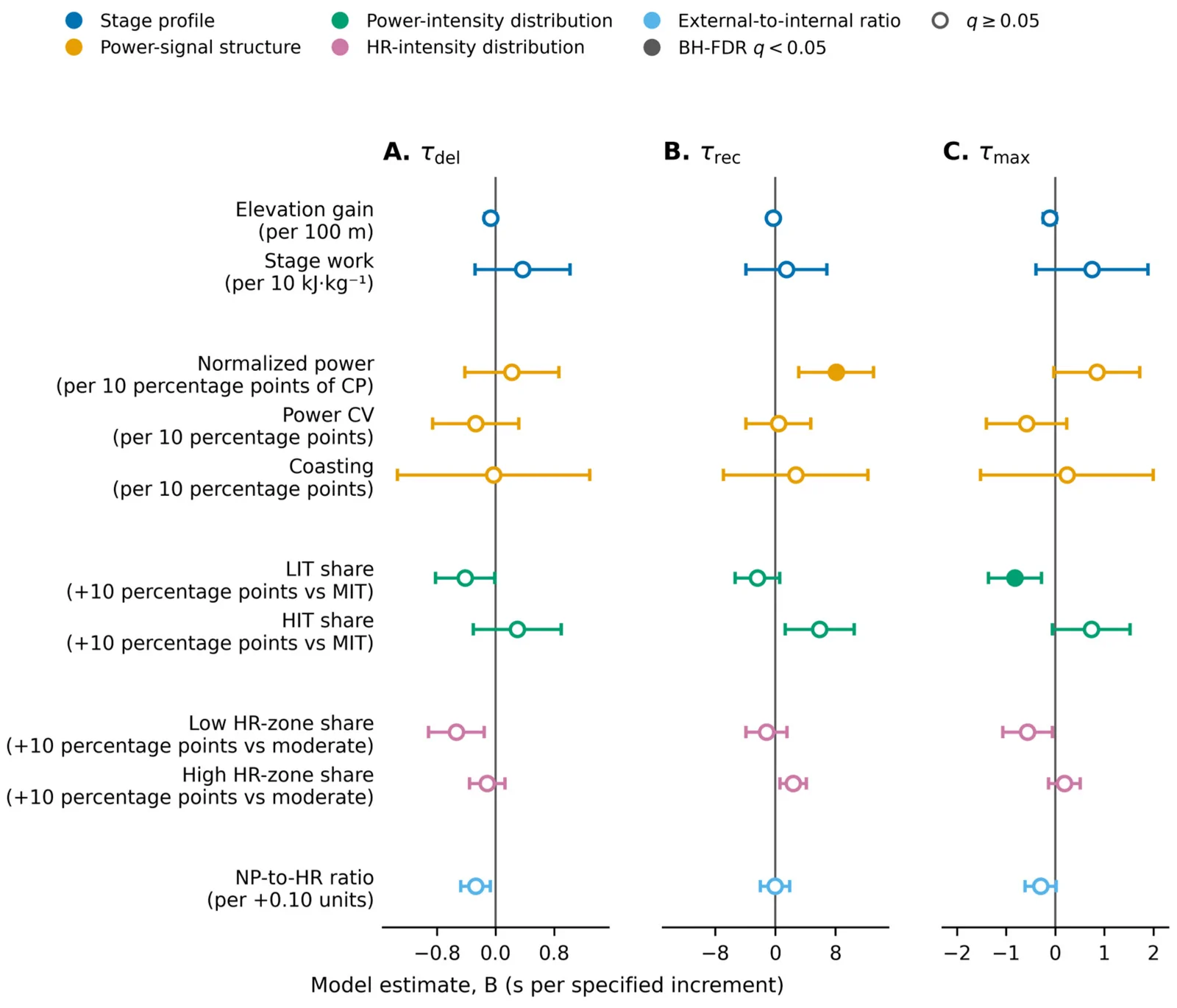
Associations between stage context and model-derived HR–PO parameters. Forest plots show fixed-effect estimates (**B**) and 95% confidence intervals for τ_del_ (**A**), τ_rec_ (**B**), and τ_max_ (**C**). For power-intensity distribution, MIT was the reference component; for HR-intensity distribution, the moderate HR zone was the reference component. Composition coefficients therefore represent the estimated change associated with replacing 10 percentage points of the corresponding reference component with the indicated intensity component. Filled markers indicate associations retained after Benjamini–Hochberg false-discovery-rate correction across all 30 stage-context tests (q < 0.05); open markers indicate q ≥ 0.05.

No stage-context association with τ_del_ was retained after FDR correction. Lower τ_del_ was nominally associated with greater low-HR-zone exposure (B = −0.54 s per 10 percentage points replacing moderate HR-zone exposure, 95% CI [−0.92, −0.16], *p* = 0.006, q = 0.050) and a higher NP-to-mean-HR ratio (B = −0.27 s per 0.10-unit increase, 95% CI [−0.48, −0.07], *p* = 0.008, q = 0.050). Greater LIT relative to MIT was also associated with lower τ_del_ before correction (B = −0.42 s per 10 percentage points, 95% CI [−0.82, −0.01], *p* = 0.043, q = 0.161). Elevation gain (B = −0.069 s per 100 m, 95% CI [−0.146, 0.008], *p* = 0.079, q = 0.198) and stage work (B = 0.37 s per 10 kJ·kg^−1^, 95% CI [−0.28, 1.02], *p* = 0.266, q = 0.443) were not associated with τ_del_ after correction (Figure 4; Supplementary Table S6).

Stage duration, examined separately, showed no FDR-retained association. Estimates were B = −0.59 s·h^−1^ for τ_max_ (95% CI [−1.14, −0.04], *p* = 0.037, q = 0.084), B = −0.47 s·h^−1^ for τ_del_ (95% CI [−0.95, 0.01], *p* = 0.057, q = 0.084), and B = −3.43 s·h^−1^ for τ_rec_ (95% CI [−7.33, 0.46], *p* = 0.084, q = 0.084). Cumulative prior mechanical work and Edwards’ TRIMP were strongly correlated with race progression (ρ = 0.959 and 0.945, respectively; both *p* < 0.001) and with each other (ρ = 0.940, *p* < 0.001). Corresponding VIFs were 15.16, 16.86, and 10.16, respectively, precluding interpretation of their coefficients as independent effects (Supplementary Table S7).

## 4. Discussion

This study examined stage-to-stage changes in model-derived HR–PO coupling parameters during U23 stage races and their associations with race-derived performance and stage context. τ_del_ decreased across race progression, whereas τ_rec_ and τ_max_ did not show systematic longitudinal changes. However, the magnitude of the τ_del_ decrease differed between races and was attenuated when the most mountainous stages were excluded, indicating that the longitudinal pattern was dependent on race and stage context. Performance-related differences in HR–PO trajectories were observed primarily for τ_rec_, with higher race-derived PO at 5 s and 5–30 min and better 5 min durability associated with a more negative τ_rec_ trajectory, although only the 5 s and 5 min PO interactions were also supported by cyclist-cluster bootstrap inference. At the stage level, higher relative stage intensity was associated with higher τ_rec_, whereas greater low-intensity exposure relative to moderate intensity was associated with lower τ_max_; no stage-context association was retained for τ_del_ after correction for multiple testing. Overall, these findings partially support the hypothesis and suggest that model-derived HR–PO coupling parameters are not redundant but may capture complementary and context-dependent features of the temporal relationship between external and internal load during stage racing.

### 4.1. Changes in Model-Derived HR–PO Parameters Across Stage-Race Progression

τ_del_ was the only parameter showing a systematic longitudinal change across valid stages, suggesting that the delay component of HR–PO coupling may be particularly sensitive to race progression. In the present model, τ_del_ reflects the timescale over which the delayed influence of previous PO contributes to current HR; therefore, a lower τ_del_ indicates that the estimated influence of prior PO becomes concentrated closer to the current moment [12,16]. In practical terms, this suggests that, across consecutive stages, current HR was increasingly explained by more recent PO. However, this should not be interpreted as direct evidence of faster cardiovascular responsiveness, because τ_del_ is estimated from the temporal structure of the HR and PO signals and may therefore be influenced by both changes in the HR–PO relationship and differences in the PO input between stages.

The τ_del_ decrease was context-dependent. The trajectory persisted after adjustment for model fit, PO variability, and coasting exposure, but was attenuated after adjustment for elevation gain, stage duration, and stage work and when the most mountainous stages were excluded. Moreover, τ_del_ decreased clearly in the Tour de l’Avenir but showed little evidence of change in the Giro Next Gen. These findings suggest that race progression and stage profile were intertwined with the observed longitudinal pattern. This is consistent with previous stage-race research showing that both performance capacity and internal-load responses vary across consecutive race days [10,11], while stage type and terrain substantially modify the external and internal demands imposed on cyclists [9,24]. Similarly, previous work has shown that HR responses depend on exercise intensity, recent workload history, and individual cardiovascular characteristics [14,25]. Therefore, the decrease in τ_del_ is best interpreted as a context-dependent longitudinal change in the model-derived HR–PO relationship rather than as a direct marker of accumulated workload or fatigue.

Conversely, τ_rec_ and τ_max_ did not show systematic longitudinal changes at the group level. τ_rec_ showed heterogeneous individual responses and was more closely associated with race-derived performance and relative stage intensity, suggesting that its variation may be more stage- and cyclist-specific than a uniform race-long process. In stage racing, stage profile, intensity distribution, tactics, and team roles vary substantially between consecutive days [9,24], which may obscure a uniform longitudinal trend even when τ_rec_ varies between cyclists or stage contexts. Nevertheless, its limited within-stage stability warrants caution in interpreting these associations. In addition, because τ_max_ is derived from τ_del_ and τ_rec_ [12]; its behaviour reflects the combined variation of both parameters and should not be interpreted as an independent physiological mechanism.

### 4.2. HR–PO Parameter Trajectories and Race-Derived Performance

Although τ_rec_ did not show a systematic longitudinal change at the group level, its trajectories differed according to race-derived performance characteristics. Cyclists with higher race-derived PO showed a more negative τ_rec_ trajectory across the race at 5 s and 5–30 min, while better 5 min durability was also associated with a more negative τ_rec_ trajectory after accounting for the corresponding 5-min PO. The latter finding is relevant because race-derived PO and durability represent related but conceptually distinct aspects of performance: the former describes the highest performance level observed during competition, whereas the latter describes how well that performance is preserved as within-stage work accumulates. Their low collinearity and simultaneous inclusion in the models therefore suggest that the 5 min durability association was not simply a reflection of cyclists with greater 5 min PO also showing better preservation. These findings are consistent with the concept of durability, whereby performance differences in road cycling become more evident after substantial prior work [2,3]. From a race-performance perspective, preservation of PO over several minutes after accumulated work may be particularly relevant during climbs, prolonged attacks, or decisive selections occurring late in a stage [2,9]. However, because the PO associations extended from 5 s to 30 min, the present results do not support a specific relationship between τ_rec_ trajectories and sustained endurance performance alone. Rather, they suggest that both overall race-derived performance level and, more specifically, the preservation of 5 min PO after accumulated work covary with the longitudinal behaviour of τ_rec_. However, the strength of evidence differed markedly across these associations. Only the 5 s and 5 min PO interactions were supported both by FDR-corrected model-based inference and by cluster-bootstrap intervals excluding zero, whereas the 10 to 30 min PO interactions and the 5 min durability interaction were directionally consistent but had bootstrap intervals that included zero. Because PO and durability were defined at the cyclist–race-case level, model-based intervals for these cross-level interactions are likely to understate uncertainty, and the divergence between the two inferential approaches should therefore be regarded as informative rather than incidental.

This interpretation is partly supported by previous HR–PO modelling work, in which shorter HR response times were associated with higher 10 min time-trial PO, whereas no significant associations were observed with 1 min time-trial PO [12]. Although the present study analysed longitudinal changes in stage-level HR–PO parameters rather than absolute response times, the more negative τ_rec_ trajectories observed in higher-performing cyclists are broadly compatible with this previous finding. Nevertheless, the two designs address different questions; de Leeuw et al. related absolute response times to standardized time-trial performance, whereas the present study examined changes in model-derived parameters across variable real-world stages. This distinction is relevant because race-derived performance characteristics are embedded within the tactical and contextual demands of competition, including stage profile, positioning, team role, and race strategy [10,25]. Thus, the present findings extend rather than replicate previous evidence and do not establish that a shorter or more rapidly decreasing τ_rec_ represents superior cardiovascular function.

In the primary progression-by-performance analyses, no association was retained for τ_del_ or τ_max_ after correction for multiple testing. The endpoint sensitivity analysis yielded one FDR-retained association between higher 5 s PO and higher final τ_del_; however, this was observed in the smaller endpoint subset and was not supported by the primary longitudinal interaction analysis. Thus, the direction of these associations appears more consistent than their magnitude. Moreover, the observational design does not establish whether performance characteristics influence τ_rec_ trajectories or whether both simply covary across the race. Importantly, the HR–PO parameters and performance variables were derived from the same race files and share the underlying PO signal. These associations should therefore be interpreted as covariation within the same recordings rather than as independent validation of τ_rec_ as a performance marker, which will require confirmation against independent laboratory or field performance measures.

### 4.3. Associations Between Stage Context and Model-Derived HR–PO Parameters

The analysis of the relationship between stage demands and model parameters suggests that HR–PO parameters were associated with different dimensions of stage-level race demands. However, after correction for multiple testing, no individual stage-context descriptor was retained for τ_del_. This is noteworthy because τ_del_ showed the clearest longitudinal change across race progression, yet its stage-to-stage variation could not be robustly attributed to elevation gain, stage work, PO variability, coasting, intensity distribution, or the external-to-internal load ratio. This does not exclude an influence of stage structure, as adjustment for elevation gain, stage duration, and stage work attenuated the longitudinal τ_del_ estimate and exclusion of the most mountainous stages produced a similar attenuation. Rather, it suggests that no single measured stage descriptor adequately explained this variation. Such a pattern is consistent with the complex relationship between external load and internal physiological response during endurance exercise [26,27], and with dynamic HR modelling studies showing that HR depends on recent exercise history and intensity transitions rather than current workload alone [14,25]. Mountainous stages may impose a distinct temporal PO–HR structure, with more sustained PO during climbs and low or intermittent PO during descents, when HR may recover more slowly than PO decreases [9,24]. Thus, stage architecture may contribute to the longitudinal τ_del_ pattern through multiple interacting characteristics rather than through an identifiable effect of elevation, work, or PO variability in isolation.

By contrast, τ_rec_ appeared more closely related to the acute intensity of the stage than to accumulated race load. This interpretation is consistent with previous cycling research showing that stage type and race context markedly influence intensity, load, and power-output demands [9,24]. Higher NP relative to CP was associated with higher τ_rec_, indicating that the estimated influence of preceding PO on current HR persisted for longer during stages performed at greater relative intensity. Associations with greater high-intensity power exposure and high HR-zone exposure showed the same direction but did not survive correction for multiple testing and should therefore be interpreted cautiously. This pattern suggests that τ_rec_ may reflect how long the estimated influence of previous PO persists in the HR signal when the stage includes higher relative intensity [14,25,28]. Rather than indicating slower cardiovascular recovery directly, the finding suggests that relative stage intensity modifies the temporal HR–PO relationship captured by the model. This is broadly consistent with the rationale underlying intensity-weighted internal-load metrics, such as Edwards’ TRIMP, in which the intensity distribution of exercise provides information beyond accumulated duration alone [23,29].

τ_max_ showed fewer associations and should be interpreted as a derived composite parameter, because it represents the time lag at which previous PO has the greatest estimated influence on current HR and depends on the combined behaviour of τ_del_ and τ_rec_ [12,16]. Higher values therefore indicate that this maximal influence occurs further back in the preceding seconds. In the present study, greater low-intensity exposure relative to moderate-intensity exposure was associated with lower τ_max_. This indicates that, in stages containing a greater proportion of low-intensity riding at the expense of moderate intensity, the point of maximal estimated influence of previous PO occurred closer to the current moment. Given that τ_max_ is mathematically derived from τ_del_ and τ_rec_, however, this association should not be interpreted as evidence of a separate physiological mechanism. Rather, it likely represents the combined behaviour of the delay and decay components under different power-intensity distributions.

Finally, a methodological contribution should also be considered because HR–PO parameters are estimated from the temporal structure of each stage file. Sustained climbs, descents, coasting periods, PO variability, and the range of intensities available for model fitting may influence how the delayed and recovery components of the HR response are identified. This is consistent with dynamic HR modelling approaches showing that estimated HR responses depend on the pattern of intensity transitions and individual response characteristics [14,25,28]. Model fit actually improved across race progression, with increasing R^2^ and decreasing RMSE and MAE, and the τ_del_ trajectory persisted after adjustment for model fit together with PO variability and coasting exposure. Thus, progressively poorer model performance is unlikely to explain the longitudinal τ_del_ pattern. Nevertheless, differences in the temporal structure of the PO signal between stages remain a plausible contributor to the estimated parameters and cannot be fully separated from changes in the HR–PO relationship in the present observational design.

### 4.4. Practical Applications

This study provides proof of concept for a novel analytical approach based on model-derived HR–PO coupling parameters, but this approach is not yet ready for practical implementation in individual cyclists. At present, its potential value lies in describing changes in the temporal relationship between external and internal load across different race and stage contexts. The context dependence of the τ_del_ trajectory and the limited within-stage stability of the parameters, particularly τ_rec_, indicate that individual values should be interpreted cautiously. Instead, HR–PO coupling should be considered together with stage context, race-derived performance, terrain profile, power-intensity distribution, and model-fit quality. This approach may help coaches and practitioners characterize temporal HR–PO dynamics at the group level beyond what can be inferred from PO, HR, TRIMP, or MMP profiles alone, although independent validation is required before individual monitoring applications can be recommended.

### 4.5. Limitations

Several limitations should be acknowledged. First, the final analytical sample was modest, included only male U23 cyclists, and was derived from two specific stage races, which limits the generalizability of the findings to other competitive levels, female cyclists, and different race formats. In addition, 32 of the 55 screened cyclist–race cases were not represented in the primary analysis, mainly because the required HR or relative-PO signals were unavailable rather than because stage-level models failed the validity criteria. Although the included and excluded cyclists showed broadly similar descriptive characteristics, inclusion depended partly on the availability of sufficiently complete HR and PO recordings. Thus, some technical selection bias cannot be excluded, and the findings may be less generalizable to cyclists with less complete or consistent monitoring data.

Second, this was a retrospective field-based study using cyclists’ own devices during real competition. Therefore, differences in power-meter and HR-monitor characteristics, calibration, recording quality, and environmental conditions may have influenced the results. Such measurement variability could affect both HR–PO parameter estimation and the performance and stage-context variables derived from the same signals. A single body mass value was also used for each cyclist–race case. Moreover, HR_max_ was obtained from the maximum HR achieved during the race rather than from laboratory testing, which may have affected HR_max_-derived variables such as HR-zone distribution and Edwards’ TRIMP.

Third, the modelling and analytical approach has inherent limitations. HR–PO models were fitted at the stage level, and parameter estimates may have been influenced by the temporal structure of each race file, including climbs, descents, coasting periods, PO variability, and the range of intensities available for model fitting. Although model fit improved across race progression and the τ_del_ trajectory persisted after adjustment for model-fit indices and PO-signal structure, differences in stage signal structure may still have influenced parameter estimation. In addition, the analytical kernel and sigmoid delay function were specified a priori, and alternative functional forms were not evaluated; therefore, the estimated parameters may partly depend on the assumed model structure. Moreover, the reported R^2^ represents in-sample goodness of fit rather than external validation, and split-half analyses showed limited within-stage stability, particularly for τ_rec_. In addition, CP was estimated using a three-point 1/t model, which may introduce uncertainty in NP expressed relative to CP and the CP-based intensity zones. The model also contains no explicit term for within-stage cardiovascular drift. Consistent with this limitation, residual bias became progressively more negative from the first to the second half of stages, indicating increasing underestimation of observed HR. Thus, time-dependent changes in the HR–PO relationship during prolonged exercise may remain in the residual structure and could influence stage-level parameter estimation.

Finally, the HR–PO parameters and race-derived performance variables were obtained from the same recordings and share the underlying PO signal; their associations therefore represent covariation rather than independent validation of the HR–PO parameters against performance. Although FDR correction and robustness analyses were applied, the number of cyclists and shared race-stage contexts remained limited. The observational design does not allow race progression, accumulated load, and stage profile to be fully disentangled, with cumulative prior load being strongly collinear with race progression. Important contextual factors such as nutrition, hydration, sleep, thermal strain, psychological stress, tactical role, team strategy, and recovery status were not directly measured. In addition, race-derived PO and durability were defined at the cyclist-race-case level whereas the HR–PO parameters varied at the stage level. The corresponding progression-by-performance terms are therefore cross-level interactions estimated without a random slope for progression, which was not retained because of a near-boundary covariance structure. Model-based standard errors for these terms are consequently expected to be anticonservative, and the divergence between model-based and cluster-bootstrap intervals for several of these associations reflects this limitation. Therefore, the secondary findings should be interpreted as hypothesis-generating rather than causal or confirmatory evidence.

## 5. Conclusions

In conclusion, τ_del_ was the only model-derived HR–PO coupling parameter showing evidence of a longitudinal change across race progression during U23 cycling stage races, although this estimate did not remain below the significance threshold after correction across the three parameters (q = 0.063). The decrease also differed between races and was attenuated when the most mountainous stages were excluded, indicating that the longitudinal pattern was context dependent and could not be separated fully from stage profile. After correction for multiple testing, performance-related differences were confined primarily to τ_rec_, with higher race-derived PO and better 5 min durability associated with a more negative τ_rec_ trajectory; of these, only the 5 s and 5 min PO interactions were also supported by cyclist-cluster bootstrap inference. At the stage level, higher relative stage intensity was associated with higher τ_rec_ and greater low-intensity exposure relative to moderate intensity with lower τ_max_, whereas no stage-context association was retained for τ_del_. Overall, the three model-derived HR–PO parameters appear to provide complementary but context-dependent descriptions of temporal HR–PO dynamics rather than direct markers of fatigue or physiological adaptation. These secondary associations are hypothesis-generating and require validation against independent performance and physiological measures before individual monitoring applications can be established.

## Figures and Tables

**Table 1:** Descriptive characteristics of the longitudinal analytical samples.

Characteristic	Primary Sample	Complete Case Sample	Newly Included Cyclists	Cyclists Not Represented in the Primary Sample
Unique cyclists	19	10	9	26
Cyclist–race cases	23	10	10	32
Age (years)	20.7 ± 1.1	20.6 ± 1.1	20.8 ± 1.2	20.0 ± 1.1
Body mass (kg)	65.6 ± 6.5	63.5 ± 5.6	68.0 ± 7.0	65.8 ± 4.8
Height (cm)	179.9 ± 7.9	176.0 ± 5.2	184.3 ± 8.3	181.1 ± 4.3
CP (W·kg^−1^)	5.89 ± 0.53	5.80 ± 0.55	6.05 ± 0.57	5.85 ± 0.41
W′ (J·kg^−1^)	197 ± 79	186 ± 48	211 ± 110	167 ± 50
Normalized GC rank (%)	50.5 [13.6–69.6]	52.0 [11.0–57.5]	44.8 [23.0–72.0]	53.3 [21.3–70.3]

Note. Age, body mass, and height are summarized at the cyclist level; normalized general classification (GC) rank is summarized at the cyclist–race case level. GC rank was normalized to the number of finishers in each race, with 0% representing first place and 100% the last classified finisher. Values are mean ± SD or median [IQR], as appropriate.

**Table 2:** Robustness analyses of the longitudinal τ_del_ trajectory.

Analysis	Stage Observations	Cyclist–Race Cases	Cyclists	Estimate [95% CI]	*p* Value
Primary model	127	23	19	−1.33 [−2.45, −0.20]	0.021
10 complete cases	64	10	10	−1.50 [−2.46, −0.54]	0.002
Strict R^2^/RMSE criteria	126	23	19	−1.40 [−2.50, −0.30]	0.012
Cluster bootstrap	127	23	19	−1.35 [−1.98, −0.67] ^1^	—
AR(1) serial dependence	127	23	19	−1.05 [−1.79, −0.31]	0.005
Tour de l’Avenir	53	10	10	−2.48 [−3.59, −1.37]	<0.001
Giro Next Gen	74	13	13	−0.36 [−1.89, 1.17]	0.644
Most mountainous stages excluded	99	23	19	−0.85 [−2.56, 0.85]	0.327
Boundary τ_del_ estimate excluded	126	23	19	−1.32 [−2.40, −0.25]	0.016

Notes. Estimates represent the beginning-to-end change in τ_del_ across normalized race progression. ^1^ Cluster-bootstrap estimate and percentile 95% CI. The most-mountainous-stage sensitivity excluded Tour de l’Avenir stages 5 (121 km; 3864 m elevation gain) and 6a (42 km; 2311 m), and Giro Next Gen stage 7 (163 km; 4100 m).

## Data Availability

The data supporting the findings of this study are available from the corresponding author upon reasonable request. The analysis code used for HR–PO modelling and statistical analyses is also available from the corresponding author upon reasonable request.

## Supplementary Materials

The following supporting information can be downloaded at: https://www.mdpi.com/article/10.3390/sports14090409/s1, Figure S1: Representative time-resolved HR–PO model diagnostics for low- and high-elevation stages; Table S1: Split-half stability of model-derived HR–PO parameters; Table S2: Sensitivity of the longitudinal τ_del_ trajectory to the lookback window; Table S3: Performance-related progression interactions and robustness analyses; Table S4: Endpoint and regression-to-the-mean sensitivity analyses; Table S5: Descriptive characteristics of stage-context variables; Table S6: Stage-context associations with model-derived HR–PO parameters; Table S7: Stage-context model diagnostics and sensitivity analyses.
