# Supplementary material for: Stage-to-Stage Changes in Heart Rate–Power Output Coupling During Cycling Stage Races: Associations with Race-Derived Performance and Stage Context
Source: Sports (Basel). 2026 Sep 16;14(9):409. doi: 10.3390/sports14090409 (PMC13611333; doi:10.3390/sports14090409)
Supplement: Supplementary file 1 [file sports-14-00409-s001.zip › sports-4454555-supplementary.pdf]

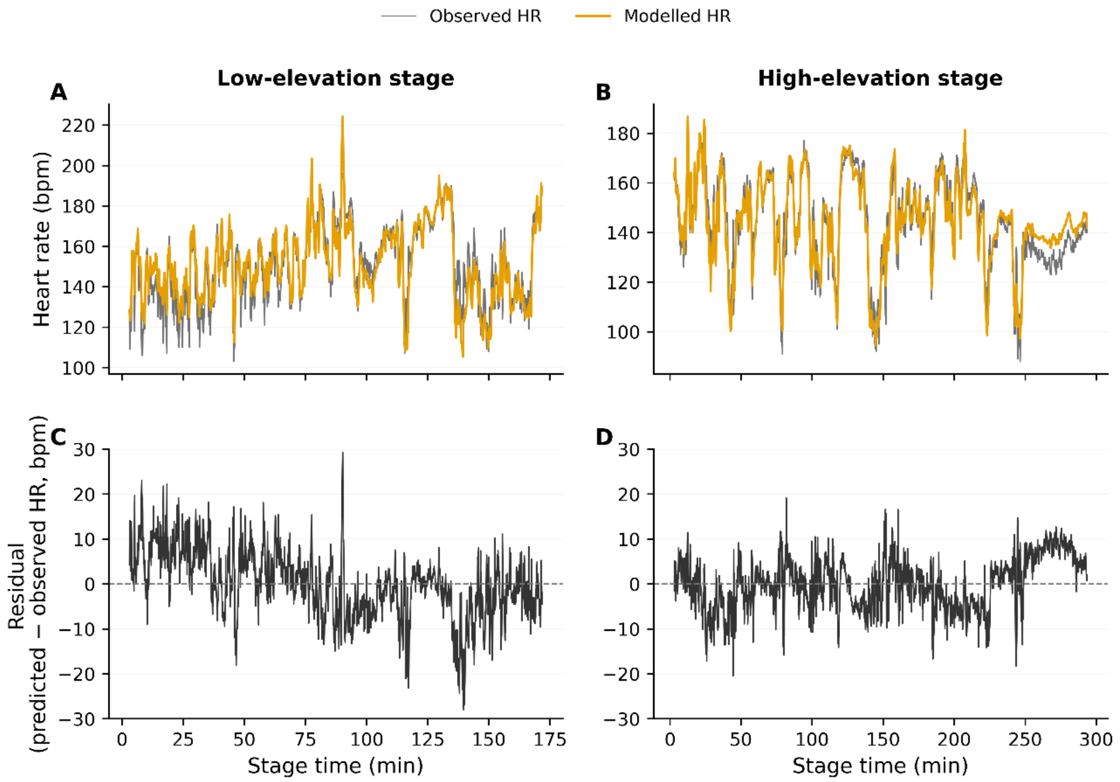

Supplementary Figure S1. Representative time-resolved HR–PO model diagnostics for low- and high-elevation stages. Observed and model-predicted heart rate (HR) and corresponding residuals (predicted − observed HR) are shown across stage time for an objectively selected low-elevation stage (elevation gain = 989 m; $R^2 = 0.84$; RMSE = 7.60 bpm) and high-elevation stage (elevation gain = 3917 m; $R^2 = 0.90$; RMSE = 5.79 bpm). Within each selected race-stage context, the cyclist-stage observation with model-fit statistics closest to the context median was selected to avoid selection based on best model fit. Panels A–B show observed and modelled HR, whereas panels C–D show the corresponding time-resolved residuals.

Supplementary Table S1. Split-half stability of model-derived HR–PO parameters

Parameter	n	First half, median [IQR]	Second half, median [IQR]	Mean difference*	Median absolute difference	Median relative difference (%)	Spearman ρ	CCC	ICC
τdel (s)	100	6.79 [5.86–7.61]	6.86 [5.60–7.62]	−0.07	1.19	18.5	0.27	0.26	0.26
τrec (s)	100	41.97 [35.10–54.55]	44.44 [38.83–55.53]	3.19	11.48	28.1	−0.06	0.01	0.01
τmax (s)	100	10.83 [9.41–12.22]	11.15 [9.89–12.46]	0.38	1.94	17.4	0.22	0.24	0.24

Note. Split-half models used the original 180-s lookback. For the second half, the 180 s preceding the midpoint were retained as lag history so that predictions began at the midpoint without resetting the HR–PO response kernel. The predefined primary model-validity criteria were maintained separately for each half, including successful model convergence, $R^2 \geq 0.30$, RMSE ≤ 12 beats·min^{-1}, and finite positive τmax (requiring τrec > 2·τdel). Of the 127 primary stage observations, 104 could be technically estimated in both halves and 100 met the predefined model-validity criteria in both halves. CCC, Lin's concordance correlation coefficient; ICC, two-way absolute-agreement, single-measure intraclass correlation coefficient [ICC(A,1)]. *Difference calculated as second half − first half.

Supplementary Table S2. Sensitivity of the longitudinal τdel trajectory to the lookback window

Lookback	Stages, n	Cyclist-race cases, n	τdel progression estimate (s)	95% CI	p value	Stage-level ICC vs. 180 s
120 s	126	23	−1.43	−2.24 to −0.63	**<0.001**	0.91
180 s	126	23	−1.40	−2.50 to −0.30	**0.012**	Reference
240 s	126	23	−1.42	−2.65 to −0.19	**0.024**	0.96

Note. Models were fitted to the identical set of 126 stage observations that met the validity criteria with all three lookback windows. Estimates represent the expected change from the beginning to the end of normalized race progression. Models included race as a fixed effect and crossed random intercepts for cyclist-race case and shared race-stage context. In the complete original 180-s sample, all 127 stages remained valid with 120 and 180 s, whereas 126 remained valid with 240 s.

Supplementary Table S3. Performance-related progression interactions and robustness analyses

Duration	Power output (W·kg⁻¹)					Durability (%)					
	B [95% CI]	p	q	Bootstrap 95% CI	LOO same direction (%)	B [95% CI]	p	q	Bootstrap 95% CI	LOO same direction (%)	Variance components*
**τdel**											
5 s	0.34 [−0.45, 1.13]	0.400	0.800	[−0.62, 1.43]	100	0.10 [−0.58, 0.79]	0.771	0.978	[−0.42, 0.65]	94.7	1.09 / 0.22 / 1.29
30 s	−0.06 [−0.76, 0.63]	0.862	0.978	[−0.97, 0.95]	89.5	0.35 [−0.32, 1.01]	0.310	0.650	[−0.13, 1.24]	100	1.20 / 0.21 / 1.29
1 min	−0.18 [−0.97, 0.61]	0.658	0.978	[−1.08, 0.58]	94.7	0.46 [−0.32, 1.23]	0.250	0.618	[−0.08, 1.06]	100	0.92 / 0.21 / 1.28
5 min	0.81 [−0.18, 1.80]	0.107	0.299	[0.03, 1.67]	100	0.99 [−0.15, 2.14]	0.090	0.286	[−2.48, 2.14]	100	1.21 / 0.22 / 1.25
10 min	0.62 [−0.07, 1.31]	0.076	0.268	[−0.05, 1.41]	100	−0.02 [−1.13, 1.09]	0.970	0.978	[−2.24, 1.15]	78.9	1.20 / 0.22 / 1.26
20 min	0.64 [−0.06, 1.35]	0.075	0.268	[−0.14, 1.48]	100	0.19 [−1.23, 1.62]	0.790	0.978	[−1.23, 2.40]	84.2	1.27 / 0.22 / 1.24
30 min	0.57 [−0.10, 1.23]	0.095	0.286	[−0.35, 1.27]	100	0.47 [−0.40, 1.34]	0.286	0.633	[−1.03, 1.71]	94.7	1.30 / 0.22 / 1.25
**τrec**											
5 s	−10.93 [−16.58, −5.29]	<0.001	0.003	[−23.07, −1.11]	100	−2.70 [−7.63, 2.22]	0.282	0.633	[−15.48, 2.67]	94.7	93.81 / 14.37 / 65.64
30 s	−6.21 [−11.44, −0.97]	0.020	0.106	[−16.14, 0.56]	100	−0.48 [−5.49, 4.53]	0.850	0.978	[−8.52, 5.09]	73.7	107.18 / 13.06 / 72.32
1 min	−7.39 [−13.49, −1.28]	0.018	0.106	[−23.58, 4.27]	94.7	−2.04 [−7.94, 3.86]	0.497	0.949	[−15.44, 7.40]	89.5	104.35 / 13.02 / 72.74
5 min	−14.20 [−21.52, −6.88]	<0.001	0.003	[−34.19, −0.52]	100	−14.96 [−23.35, −6.56]	<0.001	0.007	[−32.44, 13.73]	100	98.93 / 12.74 / 66.82
10 min	−9.00 [−14.20, −3.79]	<0.001	0.007	[−21.86, 0.41]	100	1.59 [−6.63, 9.81]	0.704	0.978	[−11.29, 9.92]	94.7	101.96 / 12.49 / 69.70
20 min	−7.56 [−12.97, −2.15]	0.006	0.043	[−25.48, 1.08]	100	0.75 [−10.05, 11.56]	0.891	0.978	[−13.56, 16.40]	78.9	109.18 / 12.11 / 70.68
30 min	−7.80 [−12.90, −2.70]	0.003	0.023	[−24.26, 2.64]	100	−0.91 [−7.50, 5.68]	0.787	0.978	[−10.33, 12.32]	84.2	107.12 / 12.17 / 70.97
**τmax**											
5 s	−1.02 [−2.11, 0.06]	0.064	0.268	[−2.55, 0.59]	100	−0.13 [−1.08, 0.82]	0.789	0.978	[−1.22, 0.83]	89.5	2.50 / 0.20 / 2.43
30 s	−0.95 [−1.91, −0.00]	0.049	0.230	[−1.94, 0.23]	100	0.25 [−0.66, 1.16]	0.588	0.978	[−0.68, 1.20]	100	3.29 / 0.20 / 2.40
1 min	−0.89 [−2.00, 0.23]	0.120	0.314	[−2.43, 0.47]	100	0.05 [−1.03, 1.13]	0.933	0.978	[−1.31, 1.38]	78.9	3.04 / 0.21 / 2.46

Duration	Power output (W·kg⁻¹)					Durability (%)					Variance components*
	B [95% CI]	p	q	Bootstrap 95% CI	LOO same direction (%)	B [95% CI]	p	q	Bootstrap 95% CI	LOO same direction (%)	
5 min	−0.39 [−1.79, 1.02]	0.589	0.978	[−2.45, 1.44]	94.7	−0.17 [−1.79, 1.45]	0.839	0.978	[−2.45, 2.78]	89.5	2.85 / 0.18 / 2.50
10 min	−0.20 [−1.18, 0.78]	0.692	0.978	[−1.41, 1.13]	94.7	0.26 [−1.30, 1.82]	0.741	0.978	[−2.01, 1.65]	94.7	3.21 / 0.18 / 2.51
20 min	0.01 [−1.00, 1.03]	0.978	0.978	[−1.82, 1.32]	63.2	−0.06 [−2.09, 1.97]	0.954	0.978	[−2.07, 2.71]	68.4	3.38 / 0.19 / 2.51
30 min	−0.03 [−0.99, 0.92]	0.943	0.978	[−1.59, 1.54]	73.7	−0.07 [−1.30, 1.17]	0.916	0.978	[−1.69, 1.69]	78.9	3.36 / 0.19 / 2.51

Note. B represents the additional beginning-to-end change in the corresponding model-derived HR–PO parameter associated with a 1-SD higher performance value. Power output was expressed relative to body mass ($W \cdot kg^{-1}$), whereas durability (%) was calculated as the normalized area under the curve of the percentage change in relative power across 0–60 $kJ \cdot kg^{-1}$ of accumulated work, with higher values indicating better preservation of performance. Power output and durability were entered simultaneously in each model. Models included race, the race-by-progression interaction, and crossed random intercepts for cyclist-race case and shared race-stage context. All models included 127 stage observations from 23 cyclist-race cases, corresponding to 19 unique cyclists and 13 shared race-stage contexts, converged successfully, and showed no near-zero random-effect variances. q values were obtained using Benjamini–Hochberg false-discovery-rate correction across all 42 progression-by-performance interactions. Bootstrap 95% CIs were percentile-based and obtained from 2,000 resamples clustered by unique cyclist, with all cyclist-race cases from the same cyclist resampled together. LOO indicates the percentage of leave-one-unique-cyclist-out analyses in which the coefficient retained the direction of the full-sample estimate. *Variance components are reported as cyclist-race case/shared race-stage/residual variance.

Supplementary Table S4. Endpoint and regression-to-the-mean sensitivity analyses

Parameter	Duration	Power output (W·kg⁻¹)			Durability (%)			R^2 / adjusted R^2
		B [95% CI]	p	q	B [95% CI]	p	q	
τdel	5 s	1.13 [0.38, 1.88]	0.007	0.046	0.54 [−0.03, 1.11]	0.062	0.216	0.56 / 0.40
	30 s	0.76 [−0.39, 1.91]	0.176	0.516	0.43 [−0.00, 0.85]	0.051	0.216	0.46 / 0.26
	1 min	−0.11 [−1.22, 1.00]	0.826	0.988	−0.51 [−2.14, 1.12]	0.508	0.712	0.36 / 0.13
	5 min	0.85 [−0.58, 2.28]	0.221	0.516	1.11 [−0.62, 2.84]	0.188	0.438	0.44 / 0.24
	10 min	0.33 [−0.63, 1.30]	0.469	0.822	0.23 [−1.19, 1.65]	0.729	0.851	0.39 / 0.16
	20 min	−0.07 [−0.87, 0.73]	0.847	0.988	1.03 [−1.44, 3.51]	0.380	0.666	0.41 / 0.20
	30 min	−0.01 [−0.99, 0.98]	0.988	0.988	−0.06 [−1.52, 1.39]	0.926	0.926	0.35 / 0.11
τrec	5 s	−5.45 [−21.13, 10.24]	0.464	0.588	1.05 [−3.81, 5.92]	0.646	0.646	0.49 / 0.31
	30 s	−3.60 [−15.00, 7.79]	0.504	0.588	1.44 [−3.19, 6.07]	0.510	0.646	0.45 / 0.25
	1 min	1.35 [−10.42, 13.11]	0.807	0.807	1.91 [−5.51, 9.33]	0.586	0.646	0.42 / 0.20
	5 min	−7.42 [−14.43, −0.41]	0.040	0.278	−5.03 [−14.13, 4.07]	0.252	0.588	0.51 / 0.34
	10 min	−5.32 [−11.81, 1.17]	0.099	0.286	−4.32 [−14.16, 5.51]	0.357	0.625	0.57 / 0.41
	20 min	−2.04 [−5.56, 1.48]	0.231	0.404	−12.34 [−23.30, −1.38]	0.030	0.213	0.70 / 0.59
	30 min	−5.17 [−11.96, 1.61]	0.122	0.286	−5.03 [−11.40, 1.34]	0.111	0.388	0.64 / 0.51
τmax	5 s	0.20 [−1.11, 1.50]	0.749	0.874	0.80 [−0.03, 1.63]	0.056	0.206	0.70 / 0.59
	30 s	0.40 [−0.73, 1.53]	0.453	0.874	0.48 [−0.02, 0.97]	0.059	0.206	0.66 / 0.54
	1 min	0.65 [−1.24, 2.54]	0.468	0.874	0.43 [−0.90, 1.76]	0.492	0.768	0.65 / 0.52
	5 min	−0.03 [−1.02, 0.95]	0.943	0.943	0.20 [−1.46, 1.86]	0.800	0.800	0.64 / 0.50
	10 min	−0.26 [−1.37, 0.86]	0.626	0.874	−0.25 [−1.47, 0.97]	0.659	0.768	0.64 / 0.51
	20 min	−0.24 [−1.23, 0.75]	0.611	0.874	−0.90 [−4.30, 2.51]	0.576	0.768	0.68 / 0.56
	30 min	−0.50 [−1.73, 0.72]	0.389	0.874	−0.80 [−2.10, 0.51]	0.207	0.484	0.72 / 0.62

Panel B. Regression-to-the-mean diagnostics

Parameter	Baseline vs change, ρ	p	Baseline vs final, ρ	p
τdel	−0.35	0.184	0.36	0.165
τrec	−0.40	0.122	0.62	0.010
τmax	−0.35	0.184	0.59	0.017

Note. Endpoint analyses were restricted to cyclist-race cases with valid HR–PO parameter estimates at both the beginning (normalized race progression = 0) and end (normalized race progression = 1) of the race. Final parameter values were modelled as a function of the corresponding baseline parameter, power output, durability, and race, with cluster-robust standard errors calculated at the unique-cyclist level. Power output and durability were standardized using scaling derived from the full 23-case sample; therefore, B represents the difference in the final HR–PO parameter associated with a 1-SD higher performance value after adjustment for baseline and the other performance dimension. q values in Panel A were obtained using Benjamini–Hochberg correction across the seven performance durations within each parameter–performance construct. Panel B reports Spearman correlations between baseline values and beginning-to-end changes and between baseline and final values. A sufficiently pronounced negative correlation between baseline and change would be compatible with regression-to-the-mean effects; however, no such association reached statistical significance. ρ, Spearman rank correlation coefficient.

Supplementary Table S5. Descriptive characteristics of stage-context variables

Stage-context variable	n	Mean ± SD	Median [IQR]
Stage duration (h)	127	3.45 ± 0.71	3.48 [2.93–3.83]
Elevation gain (m)	127	2067.00 ± 847.09	1913.40 [1517.50–2230.90]
Stage work (kJ·kg^{-1})	127	46.27 ± 10.14	45.52 [39.42–52.48]
Normalized power (%CP)	127	76.89 ± 5.73	77.14 [73.28–80.79]
Power CV (%)	127	64.62 ± 10.17	64.91 [60.05–70.67]
Coasting (%)	127	11.97 ± 3.88	11.75 [9.10–14.34]
LIT (%)	127	51.35 ± 10.68	52.55 [46.10–58.72]
MIT (%)	127	31.59 ± 11.57	28.43 [23.63–35.51]
HIT (%)	127	17.06 ± 5.36	16.35 [13.58–20.43]
Low HR-zone share (%)	127	19.84 ± 10.98	18.54 [11.87–25.64]
Moderate HR-zone share (%)	127	33.15 ± 10.98	32.36 [25.83–40.48]
High HR-zone share (%)	127	47.01 ± 17.18	46.36 [35.36–57.25]
NP-to-HR ratio	127	1.97 ± 0.19	1.97 [1.84–2.08]

Note. All variables were available for the 127 valid stage observations included in the primary longitudinal sample. Power-intensity distribution was defined relative to critical power (CP): LIT <70% CP, MIT 70–100% CP, and HIT >100% CP. HR-intensity distribution was summarized as low (50–69% HRmax), moderate (70–79% HRmax), and high (80–100% HRmax). The NP-to-HR ratio was analysed as an external-to-internal load ratio and was not interpreted as metabolic efficiency. Values are descriptive stage-level summaries and do not account for repeated observations within cyclists.

Supplementary Table S6. Stage-context associations with model-derived HR–PO parameters

Descriptor block	Predictor	Unit	B [95% CI]	p	q	Bootstrap 95% CI	Same direction (%)	Variance components*
**τdel**								
Stage profile / external load	Elevation gain	per 100 m	−0.07 [−0.15, 0.01]	0.079	0.198	[−0.15, −0.01]	98.3	1.138 / 0.185 / 1.269
Stage profile / external load	Stage work	per 10 kJ·kg^{-1}	0.37 [−0.28, 1.02]	0.266	0.443	[−0.22, 1.01]	89.3	1.138 / 0.185 / 1.269
Power-signal structure	Normalized power	per 10 percentage points of CP	0.22 [−0.42, 0.87]	0.499	0.624	[−0.19, 0.73]	88.6	1.074 / 0.283 / 1.269
Power-signal structure	Power CV	per 10 percentage points	−0.27 [−0.86, 0.32]	0.365	0.498	[−0.77, 0.12]	93.9	1.074 / 0.283 / 1.269
Power-signal structure	Coasting	per 10 percentage points	−0.03 [−1.34, 1.29]	0.967	0.967	[−1.03, 1.26]	45.8	1.074 / 0.283 / 1.269
Power-intensity distribution	LIT share	per +10 percentage points vs MIT	−0.42 [−0.82, −0.01]	0.043	0.160	[−0.74, −0.14]	99.6	0.946 / 0.316 / 1.233

Descriptor block	Predictor	Unit	B [95% CI]	p	q	Bootstrap 95% CI	Same direction (%)	Variance components*
**τdel**								
Power-intensity distribution	HIT share	per +10 percentage points vs MIT	0.30 [−0.31, 0.90]	0.335	0.498	[−0.14, 0.83]	90.5	0.946 / 0.316 / 1.233
HR-intensity distribution	Low HR-zone share	per +10 percentage points vs moderate	−0.54 [−0.92, −0.16]	0.006	0.0503	[−0.88, −0.29]	100.0	0.812 / 0.162 / 1.279
HR-intensity distribution	High HR-zone share	per +10 percentage points vs moderate	−0.12 [−0.36, 0.13]	0.350	0.498	[−0.29, 0.09]	88.0	0.812 / 0.162 / 1.279
External-to-internal load ratio	NP-to-HR ratio	per +0.10 units	−0.27 [−0.48, −0.07]	0.008	0.0503	[−0.44, 0.03]	96.2	0.861 / 0.142 / 1.298
**τrec**								
Stage profile / external load	Elevation gain	per 100 m	−0.30 [−0.94, 0.33]	0.350	0.498	[−1.00, 0.25]	86.5	106.245 / 14.628 / 75.047
Stage profile / external load	Stage work	per 10 kJ·kg⁻¹	1.48 [−3.90, 6.87]	0.589	0.680	[−3.38, 8.08]	72.5	106.245 / 14.628 / 75.047
Power-signal structure	Normalized power	per 10 percentage points of CP	8.09 [3.11, 13.07]	0.001	0.044	[2.44, 13.81]	99.8	112.355 / 10.026 / 70.009
Power-signal structure	Power CV	per 10 percentage points	0.40 [−3.92, 4.72]	0.856	0.917	[−3.32, 2.94]	51.7	112.355 / 10.026 / 70.009
Power-signal structure	Coasting	per 10 percentage points	2.70 [−6.92, 12.33]	0.582	0.680	[−5.22, 11.91]	78.8	112.355 / 10.026 / 70.009
Power-intensity distribution	LIT share	per +10 percentage points vs MIT	−2.40 [−5.38, 0.57]	0.114	0.244	[−5.00, −0.85]	99.8	111.700 / 9.658 / 68.640
Power-intensity distribution	HIT share	per +10 percentage points vs MIT	5.89 [1.30, 10.48]	0.012	0.059	[1.09, 9.70]	98.7	111.700 / 9.658 / 68.640
HR-intensity distribution	Low HR-zone share	per +10 percentage points vs moderate	−1.17 [−3.91, 1.56]	0.400	0.521	[−3.06, 0.99]	87.1	106.227 / 15.708 / 63.918
HR-intensity distribution	High HR-zone share	per +10 percentage points vs moderate	2.37 [0.61, 4.14]	0.008	0.0503	[1.18, 3.86]	99.9	106.227 / 15.708 / 63.918
External-to-internal load ratio	NP-to-HR ratio	per +0.10 units	−0.04 [−1.99, 1.90]	0.965	0.967	[−1.86, 1.98]	55.9	112.195 / 12.728 / 75.001
**τmax**								

Descriptor block	Predictor	Unit	B [95% CI]	p	q	Bootstrap 95% CI	Same direction (%)	Variance components*
**τdel**								
Stage profile / external load	Elevation gain	per 100 m	−0.11 [−0.24, 0.02]	0.100	0.230	[−0.21, −0.01]	98.1	3.517 / 0.657 / 2.252
Stage profile / external load	Stage work	per 10 kJ·kg^{-1}	0.75 [−0.39, 1.89]	0.199	0.374	[−0.26, 1.66]	91.5	3.517 / 0.657 / 2.252
Power-signal structure	Normalized power	per 10 percentage points of CP	0.85 [−0.03, 1.72]	0.057	0.187	[0.13, 1.73]	98.8	2.593 / 0.463 / 2.265
Power-signal structure	Power CV	per 10 percentage points	−0.58 [−1.40, 0.23]	0.162	0.323	[−1.41, −0.05]	98.2	2.593 / 0.463 / 2.265
Power-signal structure	Coasting	per 10 percentage points	0.24 [−1.52, 2.00]	0.790	0.878	[−1.35, 2.01]	68.0	2.593 / 0.463 / 2.265
Power-intensity distribution	LIT share	per +10 percentage points vs MIT	−0.82 [−1.36, −0.28]	0.003	0.045	[−1.27, −0.40]	100.0	2.339 / 0.404 / 2.170
Power-intensity distribution	HIT share	per +10 percentage points vs MIT	0.73 [−0.06, 1.52]	0.068	0.187	[0.07, 1.42]	98.3	2.339 / 0.404 / 2.170
HR-intensity distribution	Low HR-zone share	per +10 percentage points vs moderate	−0.56 [−1.07, −0.06]	0.029	0.124	[−0.90, −0.33]	100.0	2.294 / 0.152 / 2.259
HR-intensity distribution	High HR-zone share	per +10 percentage points vs moderate	0.19 [−0.13, 0.51]	0.254	0.443	[−0.02, 0.43]	96.4	2.294 / 0.152 / 2.259
External-to-internal load ratio	NP-to-HR ratio	per +0.10 units	−0.30 [−0.61, 0.02]	0.065	0.187	[−0.57, 0.08]	93.8	2.770 / 0.151 / 2.459

Note. B represents the stage-level difference in the corresponding model-derived HR–PO parameter for the predictor increment shown, adjusted for race progression, race, the race-by-progression interaction, and the other descriptors in the same block. MIT and the moderate HR zone were the omitted reference components in the power- and HR-intensity distribution models, respectively. q values are Benjamini–Hochberg adjusted across all 30 prespecified stage-context coefficients. Bootstrap 95% CIs are percentile intervals from 2,000 unique-cyclist cluster resamples; all analyses completed 2,000/2,000 successful resamples. All models included 127 stages, 23 cyclist-race cases, 19 unique cyclists, and 13 shared race-stage contexts and converged without near-zero random-effect variances. *Variance components: cyclist-race case / shared race-stage / residual. Rows retained after global FDR correction ($q < 0.05$) are shaded.

Supplementary Table S7. Stage-context model diagnostics and robustness analyses

Panel A. Mixed-model diagnostics

Parameter	Descriptor block	Case var.	Stage var.	Residual var.	Marginal R^2	Conditional R^2	Shapiro–Wilk p	Breusch–Pagan p
τdel	Stage profile / external load	1.138	0.185	1.269	0.231	0.623	0.024	0.422
τdel	Power-signal structure	1.074	0.283	1.269	0.227	0.626	0.031	0.328
τdel	Power-intensity distribution	0.946	0.316	1.233	0.255	0.632	0.022	0.269
τdel	HR-intensity distribution	0.812	0.162	1.279	0.280	0.591	0.010	0.334
τdel	External-to-internal load ratio	0.861	0.142	1.298	0.281	0.595	0.052	0.245
τrec	Stage profile / external load	106.245	14.628	75.047	0.022	0.625	<0.001	0.104
τrec	Power-signal structure	112.355	10.026	70.009	0.093	0.670	<0.001	0.083
τrec	Power-intensity distribution	111.700	9.658	68.640	0.093	0.672	<0.001	0.162
τrec	HR-intensity distribution	106.227	15.708	63.918	0.125	0.699	<0.001	0.051
τrec	External-to-internal load ratio	112.195	12.728	75.001	0.012	0.629	<0.001	0.119
τmax	Stage profile / external load	3.517	0.657	2.252	0.122	0.692	<0.001	0.614
τmax	Power-signal structure	2.593	0.463	2.265	0.187	0.654	<0.001	0.034
τmax	Power-intensity distribution	2.339	0.404	2.170	0.229	0.660	<0.001	0.347
τmax	HR-intensity distribution	2.294	0.152	2.259	0.233	0.632	<0.001	0.692
τmax	External-to-internal load ratio	2.770	0.151	2.459	0.152	0.612	<0.001	0.675

Panel B. Stage-duration sensitivity analysis

Parameter	B [95% CI] per 1 h	p	q	Case var.	Stage var.	Residual var.
τdel	−0.47 [−0.95, 0.01]	0.057	0.084	1.096	0.179	1.268
τrec	−3.43 [−7.33, 0.46]	0.084	0.084	111.087	12.445	72.871
τmax	−0.59 [−1.14, −0.04]	0.037	0.084	2.984	0.134	2.410

Panel C. Variance-inflation factors for stage-context predictors

Descriptor block	Predictor	VIF
Stage profile / external load	Elevation gain	4.44
Stage profile / external load	Stage work	4.58
Power-signal structure	Normalized power	1.34
Power-signal structure	Power CV	3.08
Power-signal structure	Coasting	1.74
Power-intensity distribution	LIT share	2.04
Power-intensity distribution	HIT share	1.30
HR-intensity distribution	Low HR-zone share	2.62
HR-intensity distribution	High HR-zone share	2.82
External-to-internal load ratio	NP-to-HR ratio	1.08
Duration sensitivity	Stage duration	1.29

Panel D. Cumulative prior-load collinearity diagnostics

Diagnostic	Comparison / term	Value	p
Spearman ϱ	Race progression vs previous mechanical work	0.959	<0.001
Spearman ϱ	Race progression vs previous Edwards' TRIMP	0.945	<0.001
Spearman ϱ	Previous mechanical work vs previous Edwards' TRIMP	0.940	<0.001
VIF	Race progression	15.16	—
VIF	Previous mechanical work	16.86	—
VIF	Previous Edwards' TRIMP	10.16	—

Note. Panel A reports diagnostics for the 15 primary mixed-effects models. All models were fitted using REML with crossed random intercepts for cyclist-race case and shared race-stage context, converged using the L-BFGS optimizer, and showed neither near-zero random-effect variances nor singular/boundary warnings. Panel B shows the prespecified stage-duration sensitivity analysis; q values reflect Benjamini–Hochberg correction across the three duration coefficients. Panel C reports VIFs for the stage-context predictors only; structural race and progression terms are omitted for presentation. Panel D shows why cumulative previous work and previous Edwards' TRIMP were not interpreted as independent stage-context effects: both were strongly associated with race progression and with each other, resulting in high joint-model VIFs.
